# Understanding mechanism driven regioselectivity in zirconium-catalysed hydroaminoalkylation: homoallylic amines from conjugated dienes†

**DOI:** 10.1039/d4sc00636d

**Published:** 2024-06-12

**Authors:** Erick Nuñez Bahena, Kimia Hosseini, Sheila Galván Curto, Laurel L. Schafer

**Affiliations:** a Department of Chemistry, University of British Columbia 2036 Main Mall Vancouver British Columbia Canada V6T 1Z1 schafer@chem.ubc.ca

## Abstract

The unexpected 4,1-hydroaminoalkylation of dienes provides selective access to linear homoallylic amines by zirconium catalysis. This switch from the traditional branched preferred regioselectivity to selective linear product formation using this early transition metal can be attributed to π-allyl intermediates. The reactivity of these isolated intermediates on a sterically accessible and coordinatively flexible chelating bis(ureate) Zr(iv) complex confirmed reversible C–C bond formation in hydroaminoalkylation catalysis.

## Abstract 2

A change in regioselectivity for group 4 catalysed hydroaminoalkylation gives linear homoallylic amines from conjugated dienes and amines. Isolated reaction intermediates provide mechanistic insight and evidence for reversible C–C bond formation.

## Graphical abstract

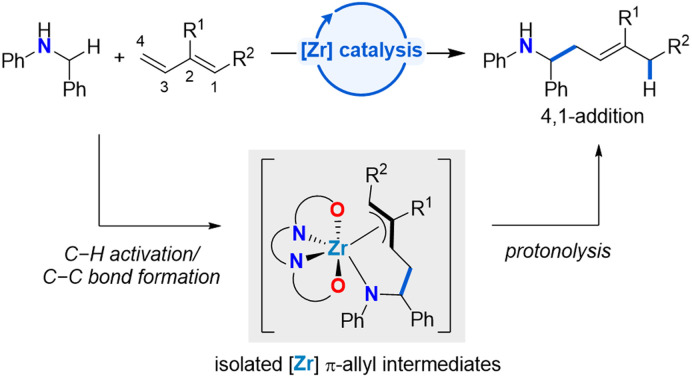

## Introduction

Homoallylic amines are valuable building blocks for the synthesis of biologically relevant compounds.^1–5^ Traditionally, metal allyl nucleophiles are added to imine electrophiles to make selectively substituted homoallylic amines.^6–15^ Here we use an Umpolung approach that features secondary benzylamines as formal carbanion nucleophiles for regioselective addition to the terminal position of 1,3-diene electrophiles to catalytically access homoallylic amines.

Dienes have been employed as abundant and easy-to-handle chemical feedstocks in reductive or borylative coupling reactions with imines to produce homoallylic amines.^16–21^ These protocols require the addition of stoichiometric reductants or borylative reagents. Recently, the development of more atom-economic catalytic methods for homoallylic amine syntheses have emerged. Late transition-metal-catalysed anti-Markovnikov hydroamination of conjugated dienes can generate homoallylic amines by regioselective C–N bond formation,^22–24^ however, more commonly allylic amines result due to preferred Markovnikov C–N bond formation *via* intermediate π-allyl metal species.^25–34^

**Scheme 1:**
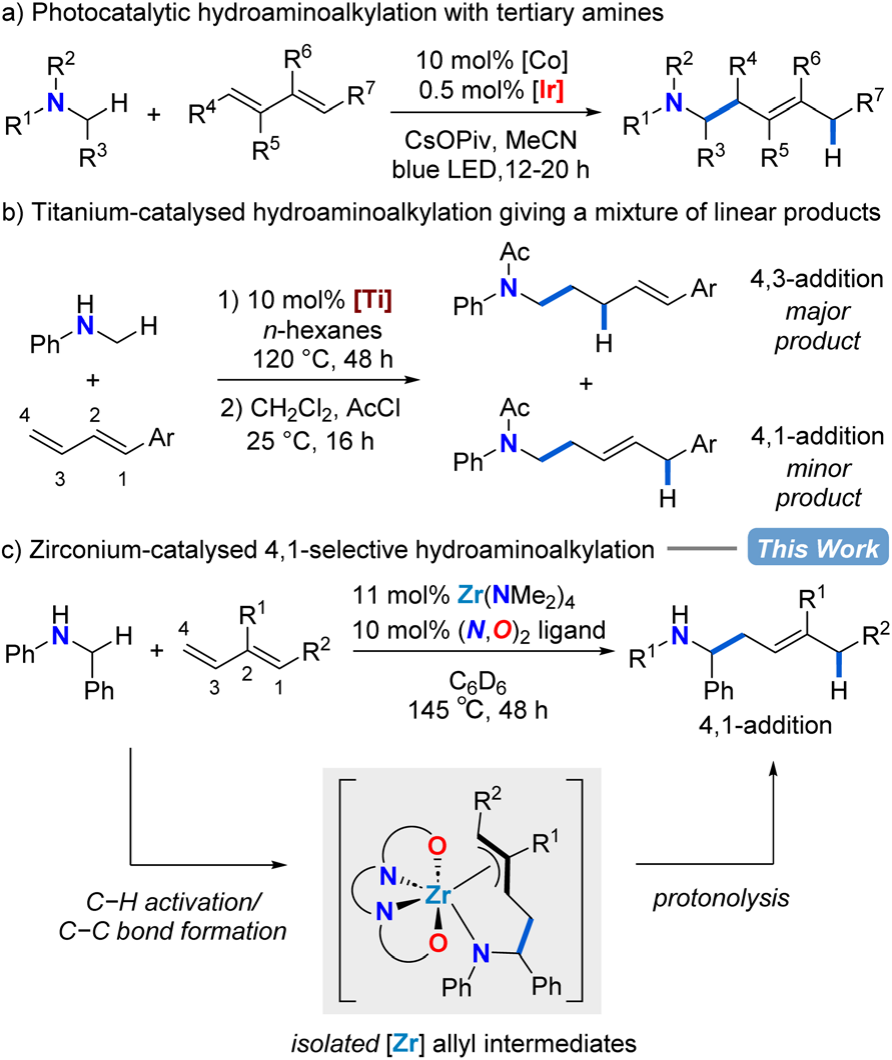
Catalytic and regioselective diene functionalization with simple amine substrates for accessing homoallylic amines.

A complementary catalytic amination reaction, hydroaminoalkylation, allows for the addition of amines directly to terminal alkenes through C–C bond formation at the α-carbon of amines.^35–37^ The hydroaminoalkylation of simple terminal alkenes with early transition metal catalysts typically affords the branched regioisomeric product.^38,39^ However, with linear regioselectivity in reactions with dienes, this transformation could be used to assemble homoallylic amines by C–C bond formation (Scheme 1).^40–44^

In 2013, using hydantoins as the nitrogen source rather than simple amine substrates, the Krische group reported a ruthenium-catalysed 4,1-hydroaminoalkylation of isoprene to give homoallylic amines.^40^ Rovis and co-workers showed that a combined cobalt and iridium photocatalytic system for diene hydroaminoalkylation can selectively access homoallylic tertiary amines as a mixture of *E*/*Z* stereoisomers (Scheme 1a).^41^ Doye has explored titanium indenyl,^42^ titanium pyridinato,^43^ and titanium mono(formamidinate)^44^ precatalysts in diene hydroaminoalkylation, although mixtures of regioisomers result (Scheme 1b). What is remarkable in these examples of titanium catalysed diene hydroaminoalkylation is that branched products are rarely observed.^42^ To date, despite these significant advances, there are neither mechanistic nor catalyst design principles to systematically favour the selective formation of a single regioisomer in diene hydroaminoalkylation.

Zirconium hydroaminoalkylation catalysis has been shown to be a synthetically useful tool for accessing selectively substituted amines with sterically demanding substrates. Substrate-controlled regioselectivity has been exploited to access linear products using vinylsilane substrates.^45,46^ Alternatively, by using L1 (Table 1) to access a sterically accessible catalytically active metal centre, new reactivity with challenging alkyne substrates was accessed.^47,48^ Given the known propensity for our zirconium catalyst with L1 to access hypercoordinate species,^48–53^ we hypothesized that this same zirconium catalyst could be used for regioselective hydroaminoalkylation of conjugated dienes that would proceed through intermediate π-allyl complexes. Herein, we show zirconium catalysed assembly of homoallylic amines *via* selective 4,1-hydroaminoalkylation of conjugated dienes. Mechanistic insights obtained from the isolation and reactivity assessment of catalytically relevant intermediates rationalizes the selective reactivity and, surprisingly, revealed a rare example of reversible alkene insertion in hydroaminoalkylation.

**Table 1:** Influence of the catalyst in the selectivity of hydroaminoalkylation of 1-phenyl-1,3-butadiene with *N*-benzylaniline

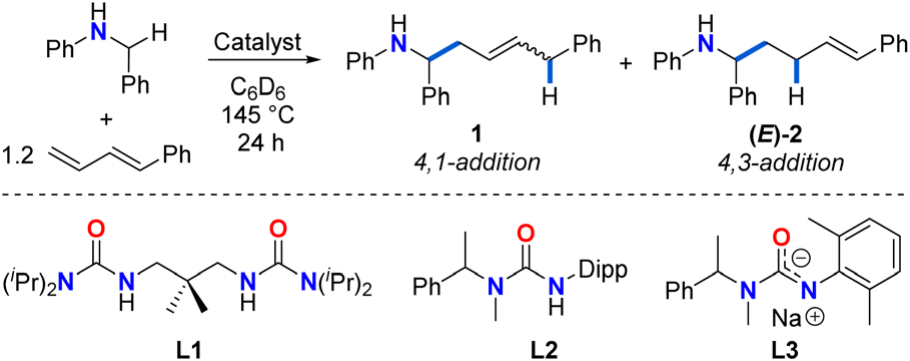				
Entry	Catalyst	1 + (*E*)-2 yield^a^ (%)	1 : (*E*)-2 ratio^a^	*E*/*Z*1 ratio^a^
1	11 mol% Zr(NMe_2_)_4_	20	85 : 15	70 : 30
2	11 mol% Zr(NMe_2_)_4_ + 10 mol% L1	57	80 : 20	91 : 9
3	11 mol% Ti(NMe_2_)_4_	44	13 : 87	52 : 48
4	10 mol% Ti(NMe_2_)_4_ + 10 mol% L2	50	>1 : 99^b^	ND^b^
5	10 mol% Ta(CH_2_SiMe_3_)_3_Cl_2_ + 10 mol% L3	No reaction	NA	NA

a Yields and ratios determined by ^1^H NMR spectroscopy using 1,3,5-trimethoxybenzene as a standard. Yields are reported as combined yields of 1 + (*E*)-2 to maintain consistency with previous reports of diene hydroaminoalkylation.^42^

b Only traces of product 1 were observed by GC-MS. The integration of signals associated with 1 was small and unreliable in GC-MS to determine *E*/*Z* ratio.

## Results and discussion

Initial investigations focused on catalyst screening for the hydroaminoalkylation of 1-phenyl-1,3-butadiene as a model diene substrate (Table 1). Using 11 mol% Zr(NMe_2_)_4_, the reaction of *N*-benzylaniline with 1.2 equiv. of 1-phenyl-1,3-butadiene in superheated *d*^6^-benzene (145 °C) in a J-Young NMR tube for 24 h afforded a mixture of linear addition products in poor NMR yield (20%, Table 1, entry 1), along with unreacted amine and a previously reported diene dimerization product.^54^ The 4,1-addition product 1 was significantly favoured over the 4,3-addition product (*E*)-2 (1/(*E*)-2 ratio = 85 : 15, 1*E*/*Z* ratio = 70 : 30).

Performing this reaction under the same conditions in the presence of 10 mol% proligand L1 (Table 1, entry 2), increased the overall yield of the reaction (57%), and retained the regioselectivity (1/(*E*)-2 ratio = 80 : 20; 1*E*/*Z* ratio = 91 : 9). Interestingly, when 11 mol% Ti(NMe_2_)_4_ is employed as catalyst (Table 1, entry 3), the regioselectivity of the reaction is considerably inverted toward the 4,3-addition product (*E*)-2 (ratio of 1/(*E*)-2 = 13 : 87; 44% overall NMR yield), suggesting the profound effect of ionic radius. The yield of (*E*)-2 can be further increased by using a state-of-the-art ureate-titanium hydroaminoalkylation catalyst (50% yield, 1/(*E*)-2 > 1 : 99; Table 1, entry 4). This selectivity for the 4,3-addition product is consistent with what has been previously reported in other titanium-catalysed diene hydroaminoalkylation reactions.^44^ While tantalum is a leading catalyst for alkene hydroaminoalkylation,^37^ a screening of a preferred catalyst system^55^ did not result in the formation of any product (Table 1, entry 5).

**Scheme 2:**
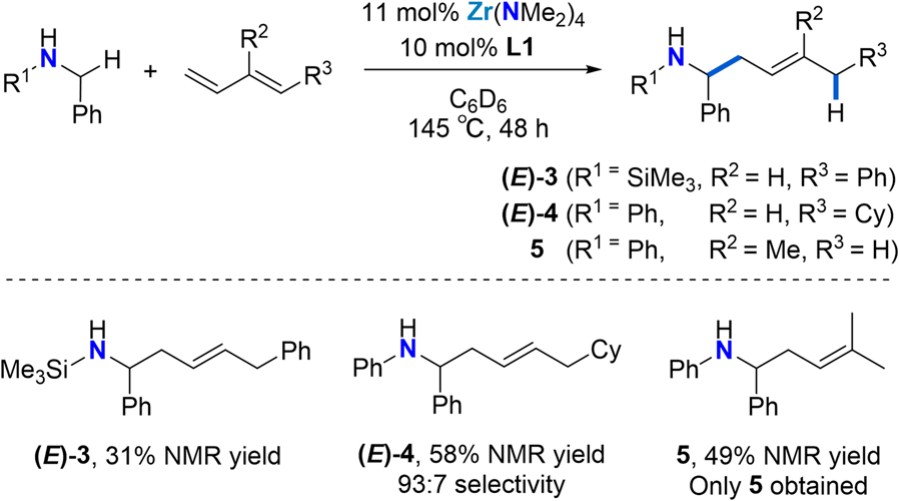
Catalytic 4,1-hydroaminoalkylation of dienes to selectively produce linear homoallylic amine products. ^a^Yields and selectivities determined by ^1^H NMR using 1,3,5-trimethoxybenzene as a standard. ^b^1.2 equiv. (*E*)-1-cyclohexyl-1,3-butadiene were employed. ^c^8–12 equiv. isoprene were employed due to substrate volatility. We did not find any variation in yield when employing >8 equiv. isoprene.

As shown in Scheme 2, the reaction is not limited to *N*-aryl substituted amines as the bulky *N*-(trimethylsilyl)benzylamine can be used as a substrate ((*E*)-3). The modest yield, as determined by ^1^H NMR spectroscopy can be attributed to diene dimerization byproduct formation. Silylamines are easily deprotected, meaning that this approach offers a strategy for the synthesis of selectively substituted homoallylic primary amines.

Next, we employed (*E*)-1-cyclohexyl-1,3-butadiene as a model alkyl-substituted diene substrate (Scheme 2). Using 11 mol% Zr(NMe_2_)_4_ and 10 mol% L1 at 145 °C for 48 h, the reaction of *N*-benzylaniline with 1.2 equiv. (*E*)-1-cyclohexyl-1,3-butadiene afforded the 4,1-addition product (*E*)-4 with a 93 : 7 selectivity in a 58% overall yield. Unreacted starting material was also observed. This result shows that the regioselectivity of these diene hydroaminoalkylation reactions is not controlled by electronic effects in the diene coupling partner.

To test if good regioselectivity is limited to 1-substituted butadienes, we tested isoprene (Scheme 2). Under the same conditions, the reaction of *N*-benzylaniline with isoprene afforded linear homoallylic amine 5 (49% yield), which forms upon selective 4,1-hydroaminoalkylation involving C–C bond formation at the less substituted terminal alkene moiety.

**Scheme 3:**
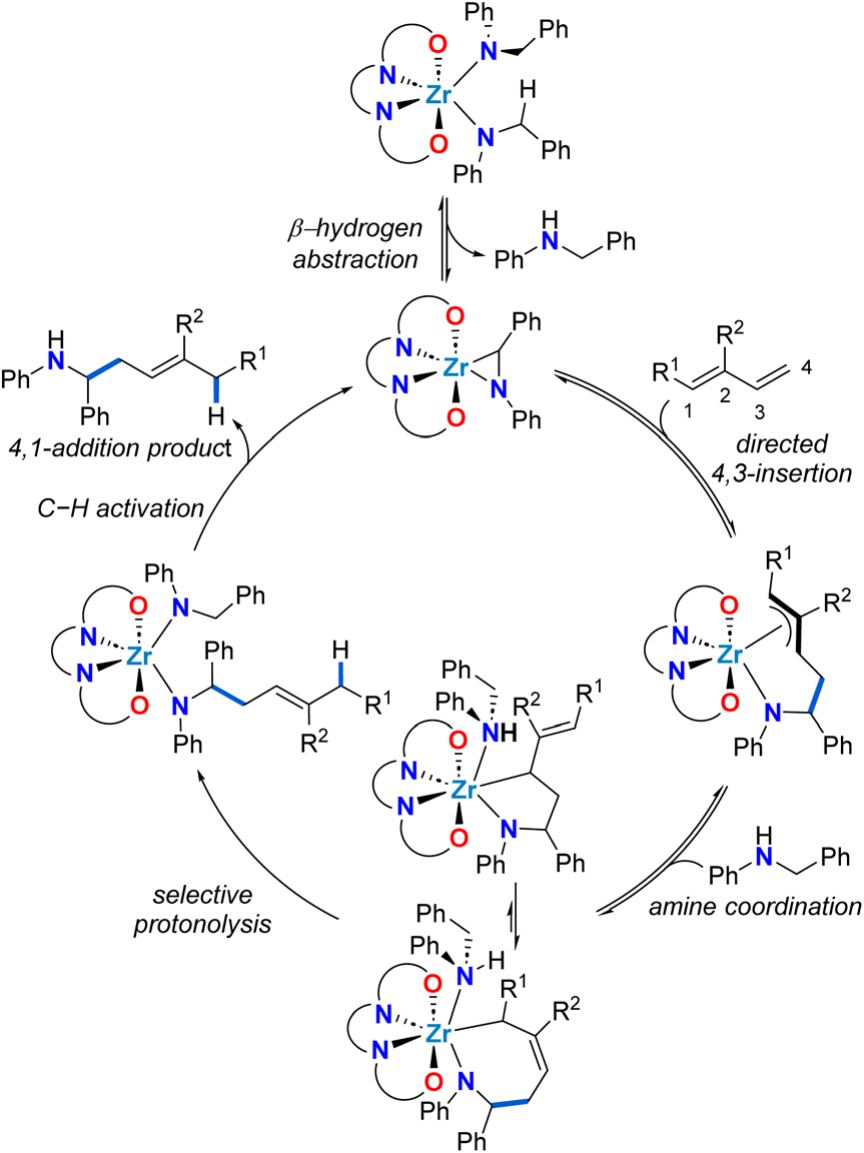
Proposed mechanistic cycle for the zirconium-catalysed 4,1-hydroaminoalkylation of conjugated dienes.

To rationalize the unexpected linear regioselectivity of these catalytic reactions, we considered the established mechanism of alkene hydroaminoalkylation (Scheme 3). As previously established, the activated C–H bond of the benzylic group of bis(*N*-benzylanilido) species promotes the formation of the required metallaaziridine catalytic intermediate.^51^ The insertion of the alkene into the M–C bond of the metallaaziridine intermediate sets the regioselectivity.^56–58^ We hypothesize that for dienes, a directing-group effect of the more electron rich C=C bond of the diene is operative. This would generate π-allyl metal intermediates that would be involved in an equilibrium between a five-membered metallacycle and a seven-membered metallacycle, that could give the 4,3 or the 4,1-addition products, respectively. The preferred 4,1-regioselectivity observed for zirconium suggests that seven-membered zirconacycles selectively undergo protonolysis and this contrasts with titanium analogues which prefer 5-membered metallacycle protonolysis to yield 4,3-addition products. This can be attributed to the larger ionic radius of Zr(iv) compared to Ti(iv), which is advantageous for accommodating both the π-allyl metal intermediates and the seven-membered metallacycle required for an associative protonolysis reaction (see Scheme 3).^48^ It should be noted that in accordance with the Curtin–Hammett principle, a turnover limiting step later in the catalytic cycle, coupled with the proposed equilibria, means that the regioselectivity is defined by the lowest energy transition state for the turnover-limiting step.

**Scheme 4:**
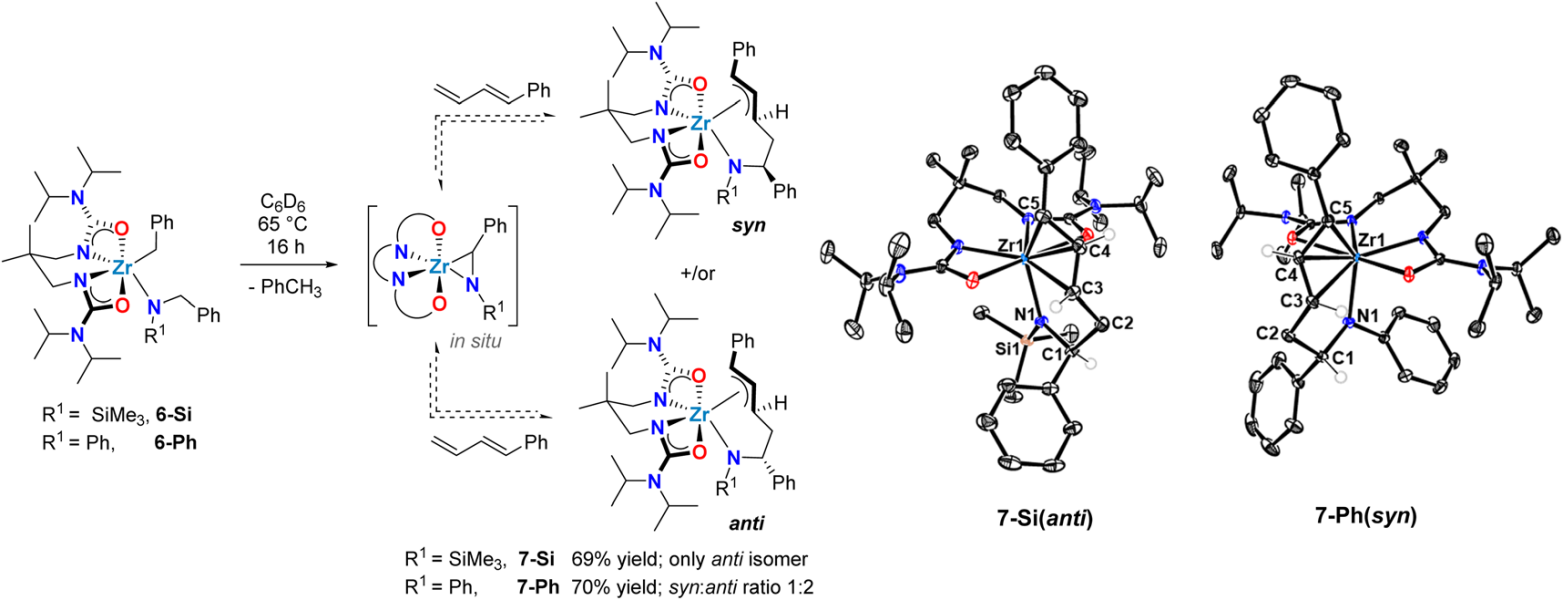
Synthesis of zirconium allyl intermediates in diene hydroaminoalkylation *via* reversible C–C bond formation involving a transient zirconaaziridine species.

Rigorous characterization of zirconium complexes supported by L1 have previously afforded insightful crystalline samples of isolated catalytic intermediates.^59^ Thus, stoichiometric reactions were pursued to isolate the putative allyl zirconium intermediates (Scheme 4). Known alkyl amido complex 6-Si ^48^ was dissolved in C_6_D_6_ and treated with 1 equiv. of 1-phenyl-1,3-butadiene (Scheme 4). Heating this reaction mixture to 65 °C gradually resulted in a change from colourless to pale yellow. Using ^1^H NMR spectroscopy, the complete disappearance of the diagnostic methylene resonances of alkylamido 6-Si was observed after 16 h, concomitant with the formation of a new zirconium complex and toluene. For the zirconium complex, the stereogenic benzylic proton α to nitrogen appears as a multiplet at *δ* = 5.73 ppm. The COSY NMR spectrum reveals a correlation between this proton and diastereotopic methylene protons at *δ* = 3.38 and 3.07 ppm. This correlation confirms regioselective C–C bond formation involving the terminal C=C double bond of 1-phenyl-1,3-butadiene. The linear regioselectivity of the insertion step, which involves the less hindered C=C double bond of 1-phenyl-1,3-butadiene, matches the observed catalytic regioselectivity (*vide supra*, Table 1).

The newly formed complex was isolated as a yellow crystalline solid in 69% yield, and single-crystal X-ray diffraction confirmed its identity as the π-allyl zirconium complex 7-Si (Scheme 4). In the solid-state, the geometry around the metal centre in 7-Si is distorted pentagonal-bipyramidal. The similar Zr–C5 (2.506(2) Å), Zr–C4 (2.518(2) Å), and Zr–C3 (2.528(2) Å) bond lengths, together with the facial coordination of the C5–C4–C3 fragment are consistent with an η^3^-allyl motif. This assignment is further corroborated by the C5–C4 (1.412(2) Å) and C4–C3 (1.390(2) Å) bond distances, which indicate delocalization of the π-electrons throughout the allylic fragment, and by the C5–C4–C3 (122.8(2)°) angle that aligns with the presence of a sp^2^-hybridized C4 centre. Notably, only a single diastereomer of 7-Si was observed by both X-ray diffraction and ^1^H NMR spectroscopy, with an *anti*-configuration of the protons bound to C1 and C3 (*vide supra*Scheme 4).

**Scheme 5:**
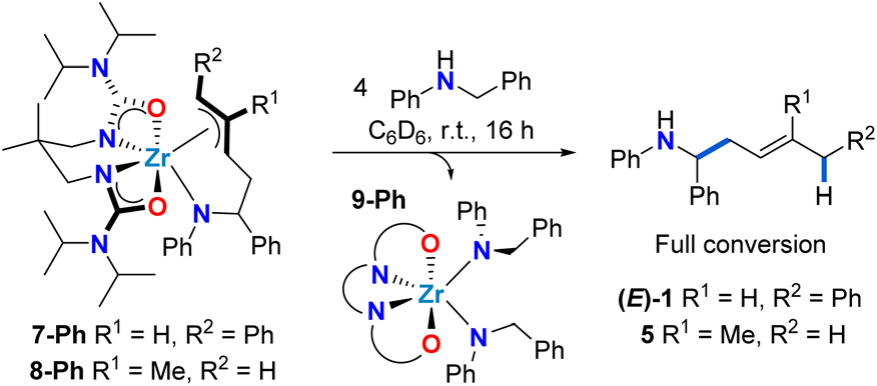
Selective release of 1,4-hydroaminoalkylation homoallylic amine product upon protonolysis of complexes 7-Ph and 8-Ph.

When the homologous *N*-arylated alkylamido complex 6-Ph was reacted with 1-phenyl-1,3-butadiene under the same conditions, the formation of two zirconium complexes was observed, consistent with the formation of diastereomers 7-Ph(*syn*) and 7-Ph(*anti*). This diastereomeric mixture was isolated in a 70% yield, and X-ray diffraction of suitable monocrystals of 7-Ph(*syn*) corroborated the identity of these π-allyl complexes. Interestingly, the diastereomer ratio varied significantly over reaction time, and reached an approximate 1 : 2 *syn* : *anti* ratio at equilibrium. This observation suggests reversible C–C bond formation, *e.g.* reactive zirconaaziridine species can be accessed from intermediate π-allyl intermediates (Scheme 4). This reversibility has been previously suggested by computational studies of alkene hydroaminoalkylation,^56–58^ and by isotopic labelling experiments in the group 5 catalyzed hydroaminoalkylation of norbornene.^60^ To corroborate the reversibility of the C–C bond-forming step, we prepared the allyl complex 7-Ph (see Scheme 5) from the reaction of *in situ* prepared 8-Ph, derived from isoprene insertion, with 5 equivalents of 1-phenyl-1,3-butadiene. Under these reaction conditions the formation of 7-Ph was observed, along with concomitant formation of free isoprene (Fig. S32†). This result confirms reversible C–C bond formation in these diene hydroaminoalkylation reactions. The reversibility of the C–C bond-forming step, coupled with a selective turnover-limiting step later in the catalytic cycle, is consistent with the observed excellent regioselectivity (*i.e.*, 4,1-addition) in these reactions.

With allyl complexes in hand, we next performed stoichiometric product release experiments (Scheme 5). Complexes 7-Ph and 8-Ph were treated with 4 equiv. of *N*-benzylaniline. Upon mixing at room temperature, the solutions turned from yellow to colourless over the course of hours. In the ^1^H NMR spectrum the clean formation of homoallylic amines (*E*)-1 and 5 was observed respectively, along with the formation of known bis(amido) complex 9-Ph.^47^ This regioselective protonolysis confirms 7-Ph and 8-Ph as reactive intermediates in catalytic hydroaminoalkylation. In addition, the ease of product release from these π-allyl intermediates, in combination with the experimentally observed reversibility of C–C bond formation suggests that the C–H activation from mixed bis(amido) species to regenerate reactive zirconaaziridine species is rate-limiting (*vide supra*Scheme 3).

## Conclusions

In conclusion, group 4 early transition metals promote a high degree of regioselectivity control (4,3-addition *vs.* 4,1-addition) in diene hydroaminoalkylation reactions by judicious choice of substrate and metal centre. This is highlighted by the hydroaminoalkylation of 1-phenyl-1,3-butadiene, where titanium catalysts exclusively afford the 4,3-addition product and zirconium-based catalysts promote the formation of the 4,1-addition product. The remarkable 4,1-selectivity of zirconium in diene hydroaminoalkylation can be exploited for accessing linear homoallylic amines from simple diene substrates. The use of a 1,3-*N*,*O*-chelating ligand in these zirconium-catalysed reactions proved to be beneficial for increasing catalytic activity and accessing regioselective product formation due to the open coordination sphere of this zirconium system. This is illustrated by the fully characterized bis(ureate)-supported zirconium allylic complexes 7-Ph/Si and 8-Ph and reversible alkene insertion, C–C bond-forming step. The facile protonolysis of π-allyl intermediates to release the 4,1-hydroaminoalkylation products exclusively points toward a rate-limiting zirconaaziridine formation from bis(amido) species. These observations rationalize the catalytic observation of the selective formation of 4,1-addition products according to the Curtin–Hammett principle.

This work shows that the catalyst design principle of generating sterically-accessible electrophilic metal centres can enable directing group effects in early transition metal chemistry, while the reversible formation of sterically demanding reactive intermediates offers efficient routes to regioselective catalytic reactivity. These ligand design concepts may be applicable to hydrofunctionalization reactions broadly.

## Data availability

Crystallographic data has been deposited at the CCDC under 2358208–2358210.

## Author contributions

LS and ENB conceived the project. LS supervised the project. ENB and KH performed the experiments and analysed data. SGC assisted in the characterization of some compounds. ENB and LS wrote the paper with contributions from all co-authors.

## Conflicts of interest

There are no conflicts to declare.

## Supplementary Material

SC-015-D4SC00636D-s001

SC-015-D4SC00636D-s002
