# Supplementary material for: Understanding mechanism driven regioselectivity in zirconium-catalysed hydroaminoalkylation: homoallylic amines from conjugated dienes
Source: Chem Sci. 2024 Jun 12;15(27):10571–6. doi: 10.1039/d4sc00636d (PMC11234830; doi:10.1039/d4sc00636d)
Supplement: SC-015-D4SC00636D-s001 [file SC-015-D4SC00636D-s001.pdf]

Electronic Supplementary Material (ESI) for Chemical Science.
This journal is © The Royal Society of Chemistry 2024

Supporting Information for:

Mechanism driven regioselectivity in zirconium-catalysed hydroaminoalkylation: homoallylic amines from conjugated dienes

*Erick Nuñez Bahena, Kimia Hosseini, Sheila Galván Curto,and Laurel L. Schafer**

Department of Chemistry, University of British Columbia, 2036 Main Mall, Vancouver, British Columbia, Canada V6T 1Z1

Table of Contents

General Considerations 2

Synthesis of Complexes 3

Catalytic experiments 5

NMR spectra 12

Crystallographic details 37

References 43

General Considerations

All air and moisture sensitive compounds were manipulated under inert N_2 atmosphere using an MBraun LABmaster glovebox or standard Schlenk techniques. Glassware and Teflon® coated magnetic stir bars were dried in a 160 °C oven for at least 4 hours prior to transferring to the glovebox or Schlenk manifold. Toluene and hexanes were passed through an activated alumina column under N_2 gas, collected in a Teflon sealed Straus flask, and sparged with N_2 for 30 minutes prior to use. Benzene-d_6 was dried over sodium metal and distilled under N_2 and collected in a Teflon sealed Straus flask prior to use. Diatomaceous earth was dried in an oven at 160 °C for at least 24 hours before transferring to the glovebox. J. Young NMR tubes (8" x 5 mm) with Teflon screw-caps were used for NMR reactions. ^{1}H NMR and ^{13}C{^{1}H} NMR spectra were collected using a Bruker 300 MHz or 400 MHz Avance spectrometer at 298 K unless otherwise noted. Chemical shifts, δ, are reported relative to the corresponding residual protio solvent in parts per million (ppm). Coupling constants, *J*, are given in Hertz (Hz). Signal multiplicity is reported using the following abbreviations: s = singlet, d = doublet, t = triplet, q = quartet, sep = septet, dd = doublet-of-doublets, qd = quartet-of-doublets, tt = triplet-of-triplets, m = multiplet, and br = broad. For NMR yield determinations, T1 relaxation times for the relevant signals were estimated using a spin-echo pulse sequence and relaxation delays were extended accordingly during ^{1}H NMR data collection. The internal standard used for quantitative ^{1}H NMR experiments was 1,3,5-trimethoxybenzene and the chemical shifts associated with it are the following: ^{1}H NMR (C_6D_6, 300 MHz) δ = 6.25 (s, 3H), 3.32 (s, 9H). For the catalytic reaction of 1-phenyl-1,3-butadiene with *N*-benzylaniline catalyzed by $Zr(NMe_2)_4$ and $Zr(NMe_2)_4$ + **L1**, 1,2,4,5-tetramethylbenzene (durene) was used as internal standard due to overlapping of the signals of 1,3,5-trimethoxybenzene with zirconium-containing species. The chemical shifts associated with durene are the following: ^{1}H NMR (C_6D_6, 300 MHz) δ = 6.86 (s, 2H), 2.09 (s, 12H). Elemental analysis (EA) was performed with a Thermo Flash 2000 Elemental Analyzer. The elemental composition values are given in percentages (%). Elemental analysis results showed consistently low carbon or nitrogen values for complexes **7-Ph** and **8-Ph**. This is likely due to the formation of zirconium oxy carbide and oxy nitride species during the combustion process as it is often observed for air and moisture sensitive early-transition-metal complexes. Single crystal X-ray diffraction data were collected using a Bruker X8 APEX or Bruker APEX DUO diffractometer. All chemicals were purchased from commercial sources and used without further purification, and those which were not already dried and stored under inert atmosphere were either dried over calcium hydride and degassed via the freeze-pump-thaw method or sublimed or dried under high vacuum before use. All liquid amines were dried over molecular sieves before degassing via the freeze-pump-thaw method and bringing them into the glovebox. All solid amines were dried and degassed in a drying tube before bringing them into the glovebox. The bis(urea) proligand,[1] the zirconium complexes **6-**

Si[2] and **6-Ph**,[3] the mono(urea) proligand used for the titanium-catalyzed reactions,[4] the sodium ureate salt,[5] and $Ta(CH_2SiMe_3)_3Cl_2$,[6] were prepared according to literature procedures.

Synthesis of Complexes

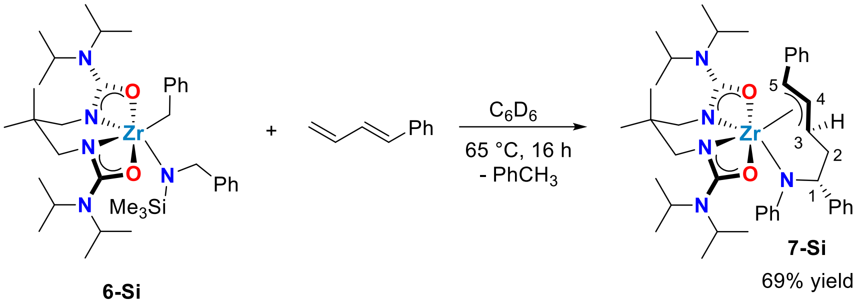

Synthesis of 7-Si: To a ~1 mL C_6D_6 solution of complex **6-Si** (0.080 g, 0.11 mmol), 1-phenyl-1,3-butadiene (0.015 g, 0.11 mmol) was added using ~0.5 mL C_6D_6 for quantitative transfer. Heating the reaction to 65 °C gradually resulted in a change from colorless to yellow. The reaction was found to be complete after 16 h by ^{1}H NMR spectroscopy. Next, the solution was transferred to a 20 mL vial and the volatiles were removed *in vacuo*. The resulting yellow powder was dissolved in ~5 mL hexanes, and the solution was cooled down to −35 °C overnight, during which time yellow crystals of complex **7-Si** (0.0583 g, 69% yield) suitable for X-ray diffraction were formed. ^{1}H NMR (C_6D_6, 400 MHz, 298 K) δ = 7.84 (m, 2H), 7.38 (m, 2H), 7.32 (m, 2H), 7.22 (m, 3H), 7.09 (m, 1H, H^4), 6.91 (m, 1H), 5.73 (d, J = 6.8 Hz, 1H, H^1), 5.70 – 5.60 (m, 1H, H^3), 4.56 (d, J = 14.5 Hz, 1H, H^5), 3.38 (m, 1H, H^2) 3.38 (m, 4H), 3.09 (m, 2H), 3.07 (m, 1H, H^2), 2.85 (d, J = 11.8 Hz, 1H), 2.75 (d, J = 11.5 Hz, 1H), 1.34 (d, J = 6.6 Hz, 6H), 1.24 (d, J = 6.6 Hz, 6H), 1.10 (d, J = 6.6 Hz, 12H), 0.70 (s, 3H), 0.58 (s, 3H), 0.14 (s, 9H, H^{SiMe3}). ^{13}C NMR (C_6D_6, 100 MHz, 298 K) δ = 172.8, 169.8, 151.3, 144.6, 144.4 (C^4), 128.2, 128.1, 127.9, 126.0, 124.3, 121.5, 108.8 (C^3), 84.4 (C^1), 83.2 (C^5), 57.4, 57.2, 47.2, 46.9, 46.4 (C^2), 36.7, 26.9, 23.4, 22.8, 22.4, 22.1, 21.3, 2.3 (C^{SiMe3}). Anal Calcd for $C_{39}H_{63}N_5O_2SiZr$: N, 9.30; C, 62.19; H, 8.43. Found: N, 9.49; C, 61.92; H, 8.07.

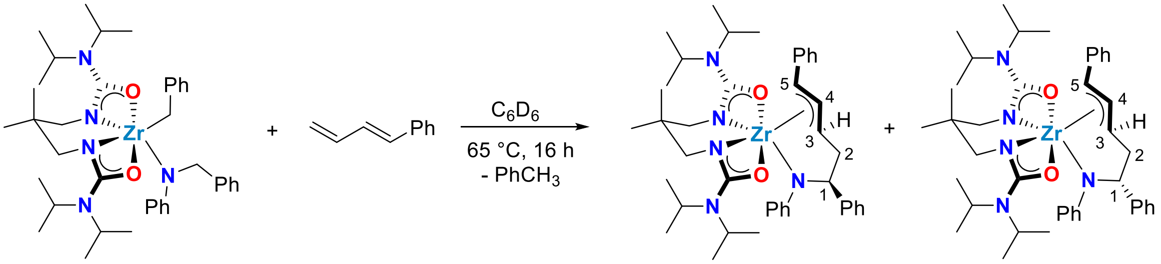

6-Ph **7-Ph(*syn*)** **7-Ph(*anti*)**

71% yield
syn:*anti* ratio 1:2

Synthesis of 7-Ph: To a ~1 mL C_6D_6 solution of complex **6-Ph** (0.181 g, 0.252 mmol), 1-phenyl-1,3-butadiene (0.035 g, 0.27 mmol) was added using ~0.5 mL C_6D_6 for quantitative transfer. Heating the reaction to 65 °C gradually resulted in a change from colorless to yellow. The reaction was found to be complete after 16 h by ^{1}H NMR spectroscopy. Next, the solution was transferred to a 20 mL vial and concentrated *in* vacuo. The resulting solution was left to stand at room temperature overnight, during which time yellow crystals of complex **7-Ph** (0.1355 g, 71% yield) were formed as a mixture of diastereomers. X-ray diffraction analysis of **7-Ph(*syn*)** confirmed the identity of these complexes. Diagnostic resonances for **7-Ph(*anti*)** (major diastereomer): ^{1}H NMR (C_6D_6, 400 MHz, 298 K) δ = 7.45 (m, 2H), 7.23 (m, 2H), 7.12 (m, 1H, H^4), 6.96 (m, 1H), 6.57 (m, 1H), 6.17 (dd, J = 11.2, 4.8 Hz, 1H, H^1), 5.63 – 5.44 (m, 1H, H^3), 4.60 (d, J = 14.3 Hz, 1H, H^5), 3.31 (dd, J = 13.1, 3.8 Hz, 1H, H^2), 3.07 (m, 1H, H^2), 1.32 (d, J = 6.6 Hz, 6H), 1.13 (d, J = 6.6 Hz, 6H), 1.06 (d, J = 6.6 Hz, 6H), 1.02 (d, J = 6.7 Hz, 6H), 0.73 (s, 3H), 0.62 (s, 3H). ^{13}C NMR (C_6D_6, 100 MHz, 298 K) δ = 147.1, 127.3, 124.4, 118.6, 112.8 (C^3), 83.4 (C^1), 82.0 (C^5), 45.6 (C^2). Diagnostic resonances for **7-Ph(*syn*)** (minor diastereomer): ^{1}H NMR (C_6D_6, 400 MHz, 298 K) δ = 7.72 (m, 2H), 7.46 (m, 2H), 7.27 (m, 1H), 7.09 (m, 1H, H^4), 6.63 (m, 1H), 6.03 (d, J = 7.4 Hz, 1H, H^1), 5.76 – 5.66 (m, 1H, H^3), 4.71 (d, J = 14.3 Hz, 1H, H^5), 3.42 (m, 1H, H^2), 3.15 (m, 1H, H^2), 2.72 (d, J = 11.6 Hz, 1H), 2.59 (d, J = 11.6 Hz, 1H), 1.25 (d, J = 6.7 Hz, 6H), 1.12 (m, 12H), 1.00 (m, 6H), 0.70 (s, 3H), 0.58 (s, 3H). ^{13}C NMR (C_6D_6, 100 MHz, 298 K) δ = 128.2, 127.9, 118.6, 112.0 (C^3), 86.9 (C^1), 83.4 (C^5), 44.4 (C^2). Overlapping resonances of both diastereomers (normalized integrations): ^{1}H NMR (C_6D_6, 400 MHz, 298 K) δ 7.45 – 6.90 (18 H), 3.51 – 2.73 (14H). ^{13}C NMR (C_6D_6, 100 MHz, 298 K) δ = 171.0, 170.0, 169.7, 169.3, 155.3, 153.0, 148.5, 144.1, 144.1, 143.0, 142.0, 128.7, 128.1, 126.3, 126.2, 121.9, 121.7, 120.0, 119.6, 83.4, 57.5, 57.1, 56.9, 47.2, 47.1, 46.8, 46.6, 36.5, 36.3, 27.1, 27.0, 22.9, 22.4, 22.3, 22.2, 21.8, 21.7, 21.6, 21.5. Satisfactory elemental analysis could not be obtained: Anal Calcd for $C_{42}H5_{9}N_5O_2Zr$: N, 9.25; C, 66.62; H, 7.85. Found: N, 9.03; C, 65.40; H, 8.20.

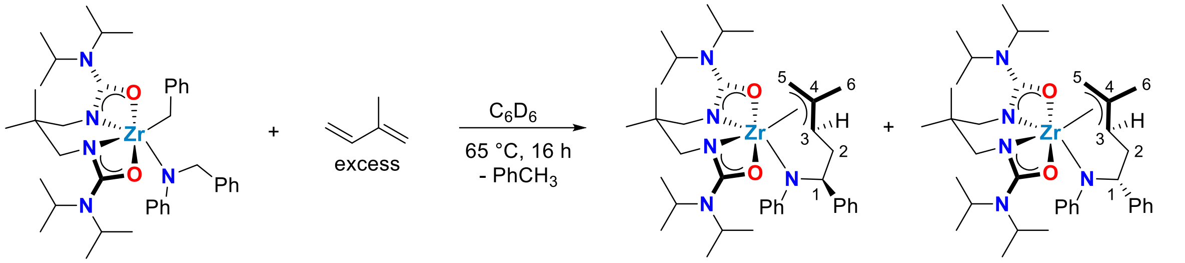

6-Ph **8-Ph(*syn*)** **8-Ph(*anti*)**

75% yield
syn:*anti* ratio 7:1

Synthesis of 8-Ph: To a ~1 mL C_6D_6 solution of complex **6-Ph** (0.273 g, 0.38 mmol), excess isoprene (> 8 equiv) was added using a micropipette. Heating the reaction to 65 °C gradually resulted in a change from colorless to yellow. The reaction was found to be complete after 16 h by ^1H NMR spectroscopy. Next, the solution was transferred to a 20 mL vial and the volatiles were removed *in vacuo*. The resulting yellow oil was redissolved in ~1 mL toluene and the solution was cooled down to −35 °C overnight, during which time yellow crystals of complex **8-Ph** (0.199 g, 75% yield) were formed as a mixture of diastereomers. These crystals were rinsed with toluene to remove impurities. X-ray diffraction analysis of **8-Ph(*syn*)** confirmed the identity of these complexes. **8-Ph(*syn*)** (major diastereomer): ^1H NMR (C_6D_6, 400 MHz, 298 K) δ = 7.51 – 7.39 (m, 2H), 7.17 – 7.02 (m, 5H), 7.02 – 6.92 (m, 3H), 6.58 (t, J = 7.1 Hz, 1H), 6.08 (t, J = 7.9 Hz, 1H, H^1), 5.40 (t, J = 6.5 Hz, 1H, H^3), 3.59 (sep, J = 6.7 Hz, 2H), 3.41 (d, J = 11.4 Hz, 1H), 3.23 (d, J = 11.0 Hz, 1H), 3.19 (sep, J = 6.7 Hz, 2H), 3.11 (m, 2H, H^2), 3.07 (d, J = 4.0 Hz, 1H, H^5) 2.99 – 2.88 (m, 2H), 2.77 (d, J = 4.0 Hz, 1H, H^5), 2.20 (s, 3H, H^6), 1.29 (d, J = 6.7 Hz, 6H), 1.15 (s, 3H), 1.12 (d, J = 6.7 Hz, 6H), 1.06 (d, J = 6.7 Hz, 6H), 0.99 (d, J = 6.6 Hz, 6H), 0.88 (s, 3H). ^{13}C NMR (C_6D_6, 100 MHz, 298 K) δ = 169.4, 168.6, 154.6 (C^4), 153.7, 147.7, 128.7, 127.4, 126.1, 118.7, 117.3, 112.3 (C^3), 80.5 (C^1), 64.8 (C^5), 58.1, 57.3, 46.8, 46.5, 41.4 (C^2), 36.1, 27.4, 24.0 (C^6), 22.7, 22.5, 22.2, 21.6. Satisfactory elemental analysis could not be obtained: Anal Calcd for $C_{37}H_{57}N_5O_2Zr$: N, 10.08; C, 63.93; H, 8.27. Found: N, 9.88; C, 62.35; H, 8.56.

Catalytic experiments

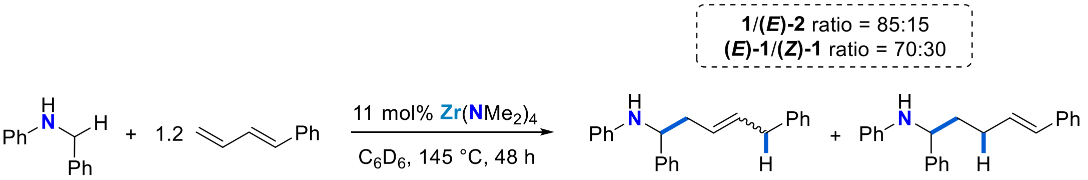

1 (***E***)-**2**

20% combined NMR yield

Hydroaminoalkylation of 1-phenyl-1,3-butadiene catalyzed by $Zr(NMe_2)_4$: To a small vial containing a solution of $Zr(NMe_2)_4$ (0.006 g, 0.02 mmol) in ~0.25 mL C_6D_6, solutions of *N*-benzylaniline (0.037 g, 0.20 mmol) and 1-phenyl-1,3-butadiene (0.030 g, 0.023 mmol) in ~0.25 mL C_6D_6 were added separately using a total of ~0.25 mL C_6D_6 for quantitative transfer. The resulting solution was transferred into a J. Young tube and a t = 0 h ^1H NMR spectrum was obtained before heating to 145 °C for 48 h. After the reaction, a solution of 1,2,4,5-tetramethylbenzene in ~0.15 mL C_6D_6 was then transferred to the reaction mixture and a ^1H NMR spectrum was obtained for NMR yield determination. The obtained yield was determined as the average yield from two duplicate experiments. The ratio of **1**/**(*E*)-2** and the ratio of **(*E*)-1**/**(*Z*)-1** was obtained from the integrations of the diagnostic benzylic resonances of each compound in the ^1H NMR spectrum. These ratios were further corroborated by GC-MS analysis. The chemical shifts for **(*E*)-1** were consistent with those previously reported in the literature.[7]

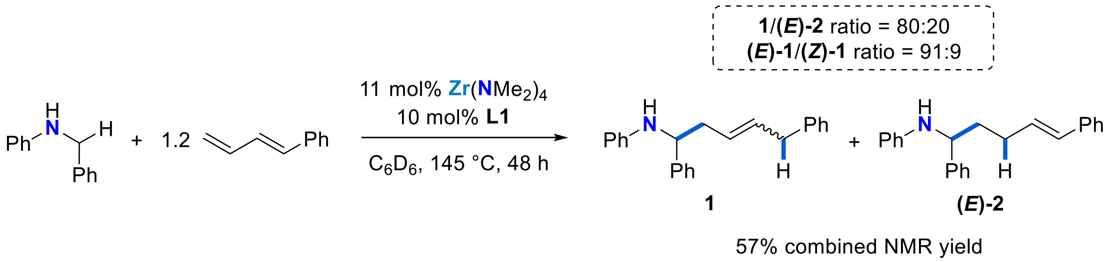

Hydroaminoalkylation of 1-phenyl-1,3-butadiene catalyzed by *in situ* formed bis(ureate) zirconium catalyst: To a small vial containing bis(urea) proligand **L1** (0.007 g, 0.02 mmol), a solution of $Zr(NMe_2)_4$ (0.006 g, 0.02 mmol) in ~0.25 mL C_6D_6 was added and mixed thoroughly to form the precatalyst. Then, solutions of *N*-benzylaniline (0.037 g, 0.20 mmol) and 1-phenyl-butadiene (0.030 g, 0.023 mmol) in ~0.25 mL C_6D_6 were added separately using a total of ~0.25 mL C_6D_6 for quantitative transfer. The resulting solution was transferred into a J. Young tube and a t = 0 h ^1H NMR spectrum was obtained before heating to 145 °C for 48 h. After the reaction, a solution of 1,2,4,5-tetramethylbenzene in ~0.15 mL C_6D_6 was then transferred to the reaction mixture and a ^1H NMR spectrum was obtained for NMR yield determination. The obtained yield was determined as the average yield from two duplicate experiments. The ratio of **1**/**(*E*)-2** and the ratio of **(*E*)-1**/**(*Z*)-1** was obtained from the integrations of the diagnostic benzylic resonances of each compound in the ^1H NMR spectrum. These ratios were further corroborated by GC-MS analysis. For characterization of the major product **(*E*)-1**, the crude reaction mixture without internal standard was quenched with ~2 mL DCM and filtered through diatomaceous earth. The volatiles were then removed *in vacuo* and the resulting residue was purified by silica column chromatography (hexanes/ethyl acetate) to obtain **(*E*)-1** in a 30% isolated yield. Isolated **(*E*)-1** contain around 5% of the **(*Z*)-1** isomer. The chemical

shifts for **(*E*)-1** were consistent with those previously reported in the literature.[7] ^{1}H NMR ($CDCl_3$, 400 MHz, 298 K) δ = 7.34 – 7.25 (m, 6H), 7.22 – 7.17 (m, 2H), 7.12 (m, 2H), 7.05 (m, 2H), 6.64 (m, 1H), 6.47 (m, 2H), 5.70 (m, 1H), 5.45 (m, 1H), 4.33 (dd, *J* = 7.8, 5.5 Hz, 1H), 4.15 (br s, 1H), 3.32 (d, *J* = 6.9 Hz, 2H), 2.58 (m, 1H), 2.46 (m, 1H).

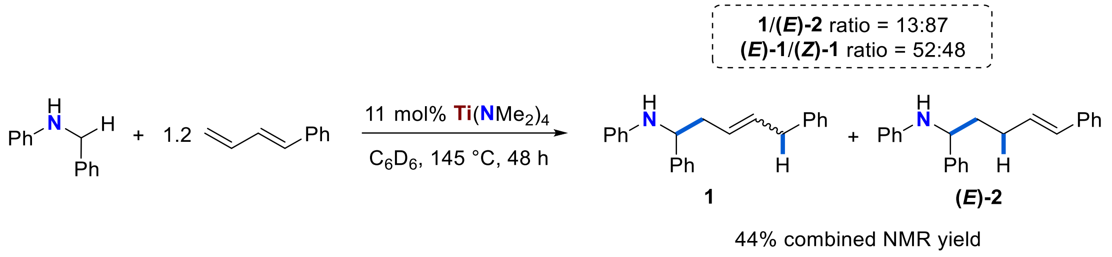

Hydroaminoalkylation of 1-phenyl-1,3-butadiene catalyzed by $Ti(NMe_2)_4$: To a small vial containing a solution of $Ti(NMe_2)_4$ (0.005 g, 0.02 mmol) in ~0.25 mL C_6D_6, solutions of *N*-benzylaniline (0.037 g, 0.20 mmol) and 1-phenyl-butadiene (0.030 g, 0.023 mmol) in ~0.25 mL C_6D_6 were added separately using a total of ~0.25 mL C_6D_6 for quantitative transfer. The resulting solution was transferred into a J. Young tube and a t = 0 h ^{1}H NMR spectrum was obtained before heating to 145 °C for 48 h. After the reaction, a solution of 1,3,5-trimethoxybenzene in ~0.15 mL C_6D_6 was then transferred to the reaction mixture and a ^{1}H NMR spectrum was obtained for NMR yield determination. The obtained yield was determined as the average yield from two duplicate experiments. The ratio of **1/(*E*)-2** and the ratio of **(*E*)-1/(*Z*)-1** was obtained from the integrations of the diagnostic benzylic resonances of each compound in the ^{1}H NMR spectrum. These ratios were further corroborated by GC-MS analysis. For characterization of the major product **(*E*)-2**, see experiment below.

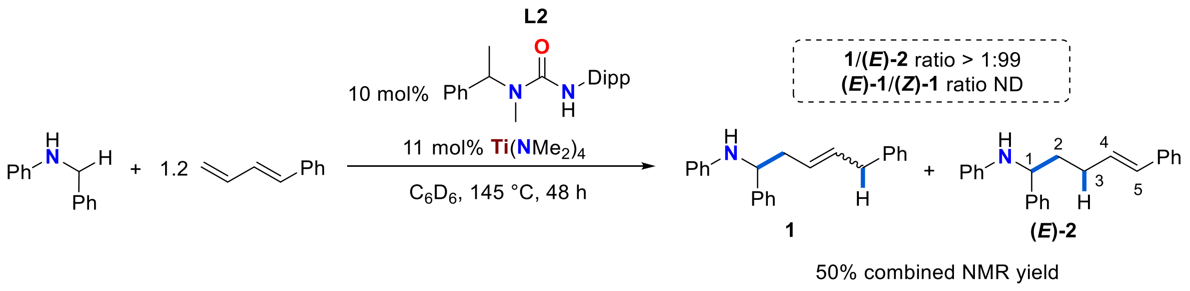

Hydroaminoalkylation of 1-phenyl-1,3-butadiene catalyzed by *in situ* formed ureate titanium catalyst: To a small vial containing the urea proligand **L2** (0.007 g, 0.02 mmol), a solution of $Ti(NMe_2)_4$ (0.005 g, 0.02 mmol) in ~0.25 mL C_6D_6 was added and mixed thoroughly to form the precatalyst. Then,

solutions of *N*-benzylaniline (0.037 g, 0.20 mmol) and 1-phenyl-butadiene (0.030 g, 0.023 mmol) in ~0.25 mL C_6D_6 were added separately using a total of ~0.25 mL C_6D_6 for quantitative transfer. The resulting solution was transferred into a J. Young tube and a t = 0 h ^{1}H NMR spectrum was obtained before heating to 145 °C for 48 h. After the reaction, a solution of 1,3,5-trimethoxybenzene in ~0.15 mL C_6D_6 was then transferred to the reaction mixture and a ^{1}H NMR spectrum was obtained for NMR yield determination. The obtained yield was determined as the average yield from two duplicate experiments. The ratio of **1**/**(*E*)-2** and the ratio of **(*E*)-1**/**(*Z*)-1** was obtained from the integrations of the diagnostic benzylic resonances of each compound in the ^{1}H NMR spectrum. These ratios were further corroborated by GC-MS analysis. For characterization of the major product **(*E*)-2**, the crude reaction mixture without internal standard was quenched with ~2 mL DCM and filtered through diatomaceous earth. The volatiles were then removed *in vacuo* and the resulting residue was purified by silica column chromatography (hexanes/ethyl acetate) to give **(*E*)-2** in a 13% isolated yield. ^{1}H NMR ($CDCl_3$, 400 MHz, 298 K) δ = 7.40 – 7.33 (m, 7H), 7.27 – 7.19 (m, 3H), 7.11 (m, 2H), 6.71 (m, 1H), 6.63 (m, 2H), 6.38 (m, 1H, H^5), 6.20 (m, 1H, H^4), 4.40 (t, J = 6.9 Hz, 1H, H^1), 2.29 (m, 2H, H^3), 2.07 (m, 2H, H^2), 1.60 (br s, 1H, NH). ^{13}C NMR ($CDCl_3$, 100 MHz) δ = 137.7, 131.0 (C^5), 129.6 (C^4), 129.3, 128.8, 128.7, 127.4, 127.2, 126.9, 126.1, 58.9[a] (C^1), 36.7[a] (C^2),29.8 (C^3). [a]Resonance found through HMBC NMR.

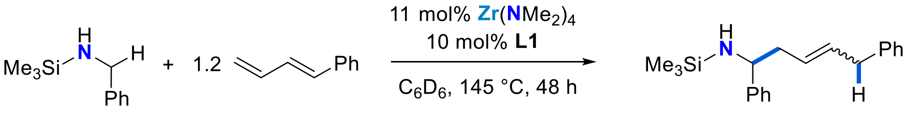

3

Hydroaminoalkylation with N-(trimethylsilyl)benzylamine catalyzed by *in situ* formed bis(ureate) zirconium: To a small vial containing bis(urea) proligand **L1** (0.007 g, 0.02 mmol), a solution of $Zr(NMe_2)_4$ (0.006 g, 0.02 mmol) in ~0.25 mL C_6D_6 was added and mixed thoroughly to form the precatalyst. Then, solutions of *N*-(trimethylsilyl)benzylamine (0.036 g, 0.20 mmol) and 1-phenyl-butadiene (0.030 g, 0.023 mmol) in ~0.25 mL C_6D_6 were added separately using a total of ~0.25 mL C_6D_6 for quantitative transfer. The resulting solution was transferred into a J. Young tube and a t = 0 h ^{1}H NMR spectrum was obtained before heating to 145 °C for 48 h. After the reaction, a solution of 1,2,4,5-tetramethylbenzene in ~0.15 mL C_6D_6 was then transferred to the reaction mixture and a ^{1}H NMR spectrum was obtained for NMR yield determination. Diagnostic resonances for **3**: ^{1}H NMR ($CDCl_3$, 400 MHz, 298 K) δ = 5.57 – 5.44 (m, 1H, olefinic CH), 5.35 (m, 1H, olefinic CH), 3.91 (m, 1H, NCHBn), 3.17 (d, J = 6.7 Hz, 2H, Bn).

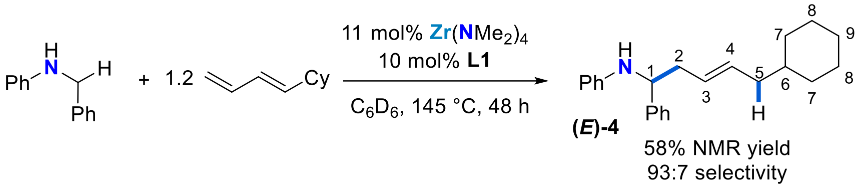

Hydroaminoalkylation of 1-cyclohexyl-1,3-butadiene catalyzed by *in situ* formed bis(ureate) zirconium catalyst: To a small vial containing bis(urea) proligand **L1** (0.007 g, 0.02 mmol), a solution of $Zr(NMe_2)_4$ (0.006 g, 0.02 mmol) in ~0.25 mL C_6D_6 was added and mixed thoroughly to form the precatalyst. After 20 min, solutions of *N*-benzylaniline (0.037 g, 0.2 mmol) and 1-cyclohexyl-1,3-butadiene (0.033 g, 0.24 mmol) were added to the precatalyst solution, using a total of ~0.25 mL C_6D_6 for quantitative transfer. Then, a t = 0 h ^1H NMR spectrum was obtained before heating to 145 °C for 48 h. After the reaction, a solution of 1,3,5-trimethoxybenzene in ~0.15 mL C_6D_6 was then transferred to the reaction mixture and a ^1H NMR spectrum was obtained for NMR yield and regioselectivity determination. The obtained yield and regioselectivity was determined as the average from two duplicate experiments. The regioselectivity toward **(*E*)-5** was obtained from the integrations of the diagnostic benzylic resonances of each compound in the ^1H NMR spectrum. For characterization of the major product **(*E*)-4**, the crude reaction mixture without internal standard was quenched with ~2 mL DCM and filtered through diatomaceous earth. The volatiles were then removed *in vacuo* and the resulting residue was purified by silica column chromatography (hexanes/ethyl acetate) to give **(*E*)-4** in a 19% isolated yield. ^1H NMR ($CDCl_3$, 400 MHz, 298 K) δ = 7.39 – 7.30 (m, 4H), 7.26 – 7.21 (m, 1H), 7.11 – 7.03 (m, 2H), 6.69 – 6.62 (m, 1H), 6.53 – 6.46 (m, 2H), 5.63 – 5.53 (m, 1H, H^4), 5.34 (m, 1H, H^3), 4.32 (dd, *J* = 8.1, 5.0 Hz, 1H, H^1), 2.60 (m, 1H, H^2), 2.44 (m, 1H, H^2), 1.93 (t, *J* = 7.0 Hz, 2H, H^5), 1.75 – 1.64 (m, 4H, H^7), 1.30 – 1.11 (m, 5H, , H^6 and H^8), 0.90 (m, 2H, H^9). ^{13}C NMR ($CDCl_3$, 100 MHz) δ = 147.5 (C^{Ph}), 143.9 (C^{Ph}), 133.5 (C^4), 129.2 (C^{Ph}), 128.7 (two overlapping resonances, (C^{Ph})), 127.0 (C^3), 126.5 (C^{Ph}), 117.5 (C^{Ph}), 113.7 (C^{Ph}), 57.7 (C^1), 42.4 (C^2), 40.7 (C^5), 38.1 (C^6), 33.3 (C^7), 33.2 (C^9), 26.5 (C^8).

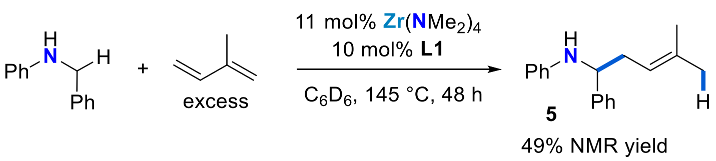

Hydroaminoalkylation of isoprene catalyzed by *in situ* formed bis(ureate) zirconium catalyst: To a small vial containing bis(urea) proligand **L1** (0.007 g, 0.02 mmol), a solution of $Zr(NMe_2)_4$ (0.006 g, 0.02 mmol) in ~0.25 mL C_6D_6 was added and mixed thoroughly to form the precatalyst. After 20 min, a solution of *N*-benzylaniline (0.037 g, 0.2 mmol) was added to the precatalyst solution, using a total of ~0.25 mL

C_6D_6 for quantitative transfer. The solution was then transferred into a J. Young tube, and excess isoprene (>8 equiv) was then added using a micropipette. A t = 0 h ^{1}H NMR spectrum was obtained before heating to 145 °C for 48 h. After the reaction, a solution of 1,3,5-trimethoxybenzene in ~0.15 mL C_6D_6 was then transferred to the reaction mixture and a ^{1}H NMR spectrum was obtained for NMR yield determination. The obtained yield was determined as the average yield from two duplicate experiments. GC-MS analysis further supported the formation of 7 as the sole regioisomer. For complete characterization of **5**, see the experiment below "Stoichiometric product release from complexes **8-Ph**".

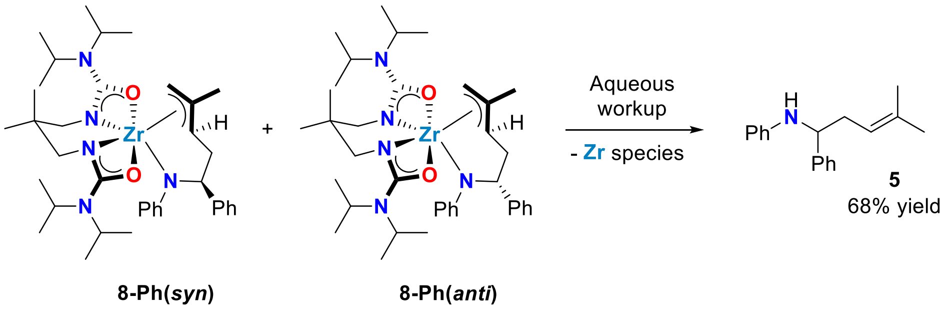

syn:*anti* ratio 7:1

Stoichiometric product release from complexes 8-Ph: A ~0.75 mL C_6D_6 solution of complex **8-Ph** (*syn*/*anti* mixture) (0.100 g, 0.14 mmol) was quenched with ~5 mL DCM and filtered through diatomaceous earth. The volatiles were then removed *in vacuo* and the resulting residue was purified by silica column chromatography (hexanes/ethyl acetate in a 50:1 volume ratio) to obtain compound **5** as a yellow oil (0.024 g, 68% yield). ^{1}H NMR ($CDCl_3$, 400 MHz, 298 K) δ = 7.40 (m, 2H), 7.35 (m, 2H), 7.26 (m, 1H), 7.12 (m, 2H), 6.69 (m, 1H), 6.56 (d, *J* = 8.0 Hz, 2H), 5.18 (m, 1H), 4.55 (br s, 1H), 4.35 (t, *J* = 6.7 Hz, 1H), 2.54 (m, 2H), 1.75 (s, 3H), 1.65 (s, 3H); ^{13}C NMR ($CDCl_3$, 100 MHz) δ = 147.3, 143.8, 135.4, 129.2, 128.6, 127.0, 126.5, 120.2, 117.7, 114.0, 58.7, 37.6, 26.0, 18.1, 1.2. HRMS(ESI) *m*/*z* Calcd for $C_{18}H_{21}N$ $[M]^+$: 251.1674; found: 251.1672.

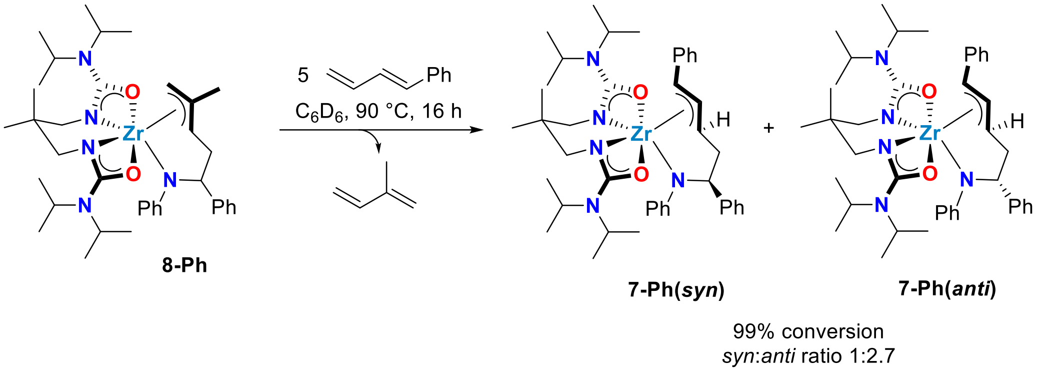

Crossover experiment between complex 8-Ph and 1-phenyl-1,3-butadiene: To a ~0.5 mL C_6D_6 solution of complex **8-Ph** (*syn*/*anti* mixture) (0.020 g, 0.029 mmol, a solution of 1-phenyl-1,3-butadiene in ~0.25 mL C_6D_6 was added and mixed thoroughly before transferring to a J.Young tube. Then, a t = 0 h ^{1}H NMR spectrum was obtained before heating to 90 °C for 16 h. After this time, the reaction was left to cool down to room temperature and a ^{1}H NMR spectrum was obtained, revealing 99% conversion to complexes 7-Ph(*syn*) and 7-Ph(*anti*) in a 1 : 2.7 ratio.

NMR spectra

Figure S1. ^{1}H NMR spectrum of 7-Si (C_6D_6, 400 MHz, 298 K).

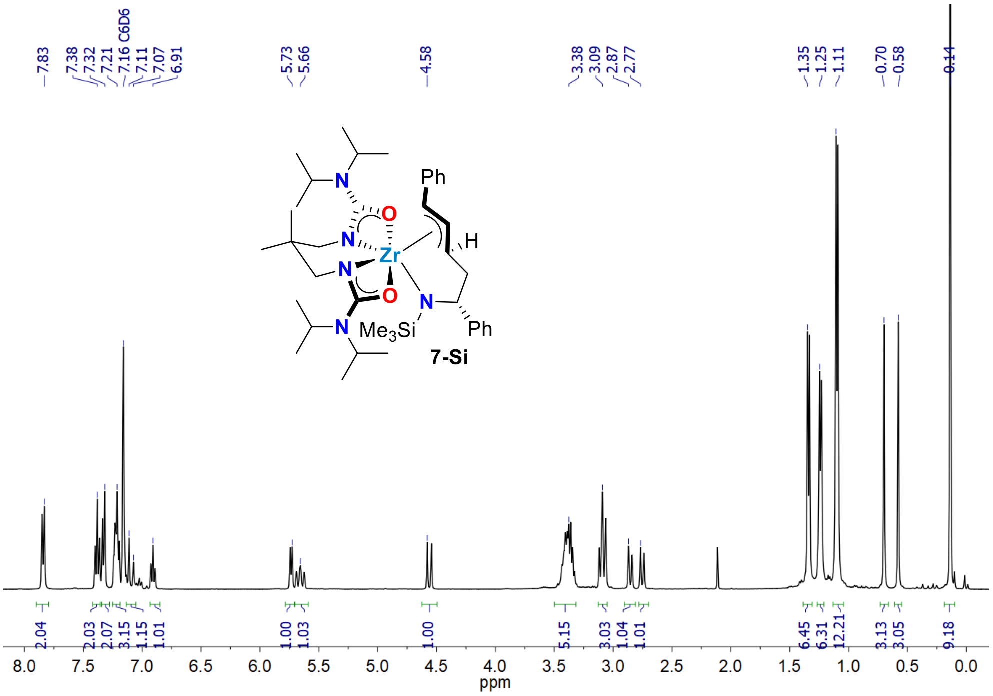

Figure S2. ^{13}C{^{1}H} NMR spectrum of 7-Si (C_6D_6, 100 MHz, 298 K).

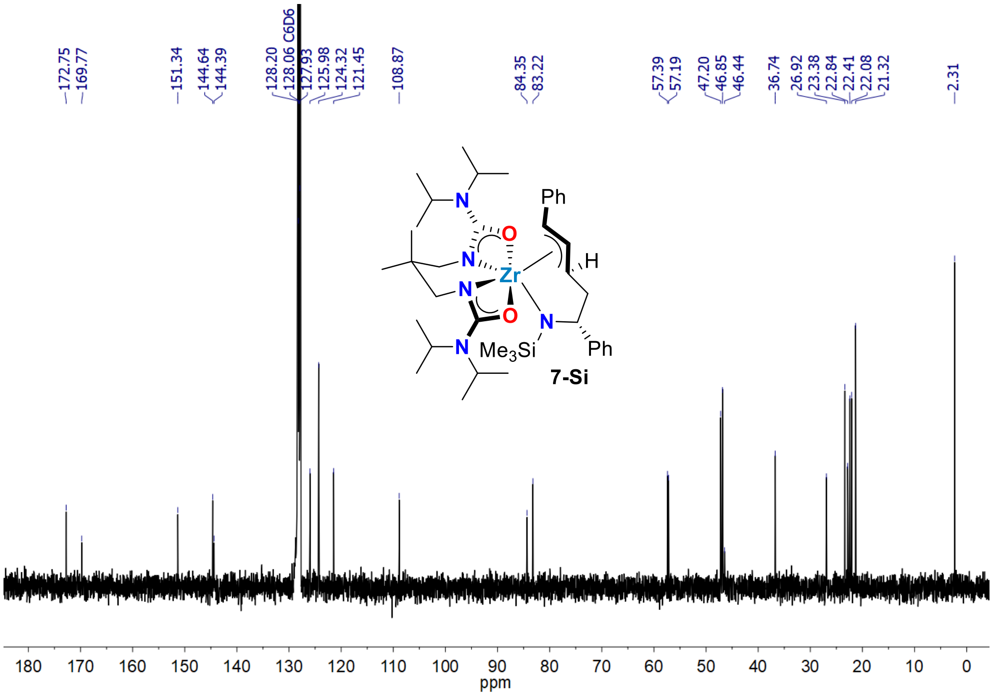

Figure S3. COSY NMR spectrum of 7-Si (C_6D_6, 400 MHz, 298 K).

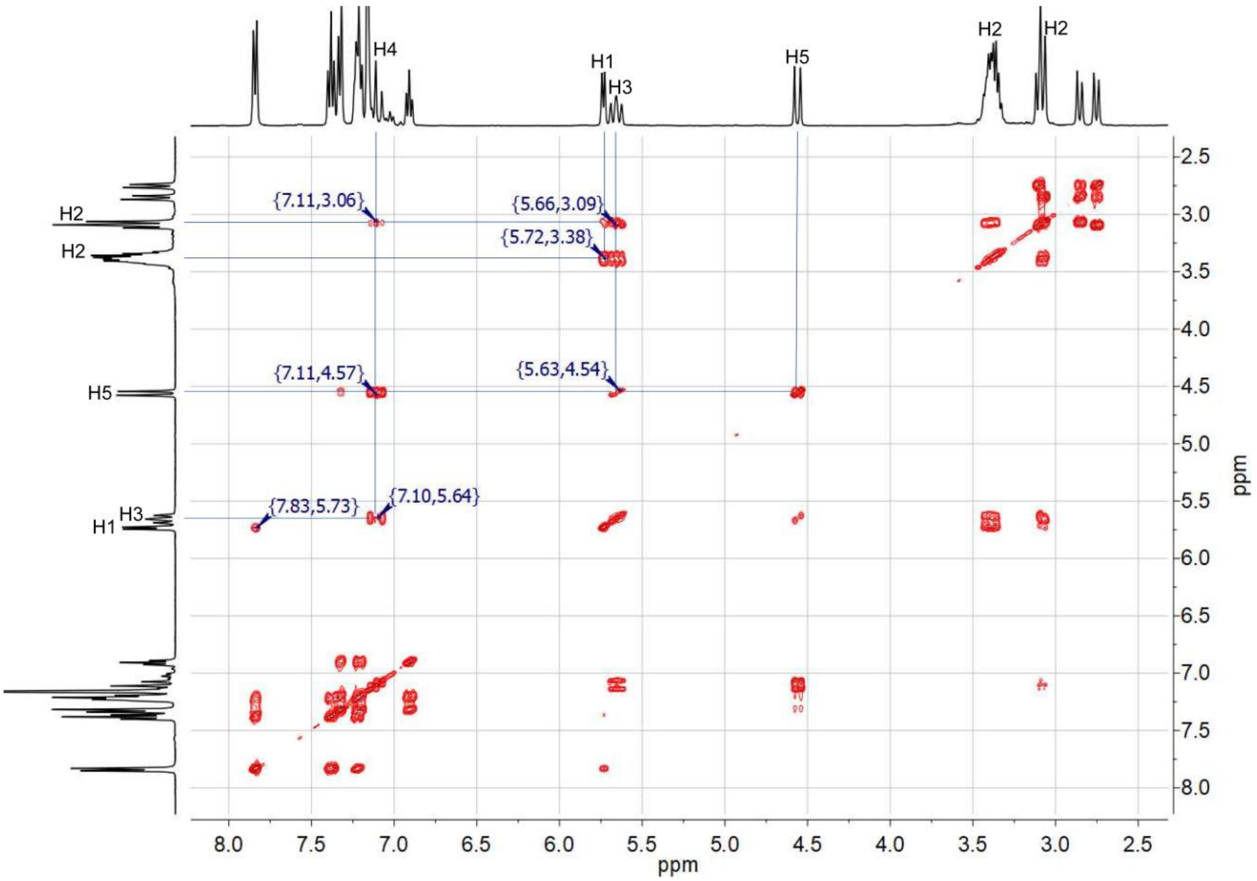

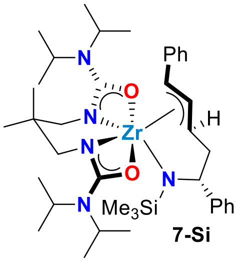

7-Si

Figure S4. HSQC NMR spectrum of 7-Si (C_6D_6, 400 MHz, 298 K).

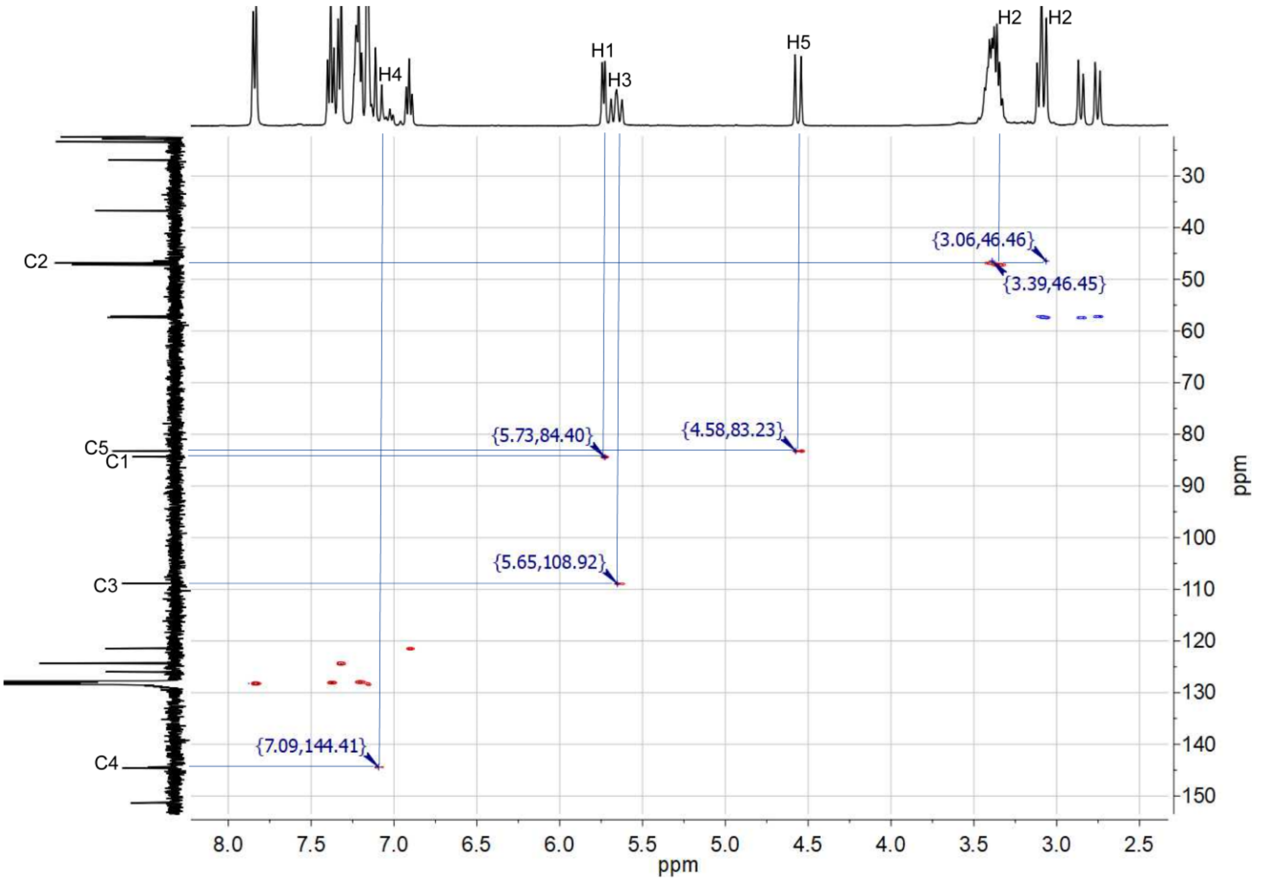

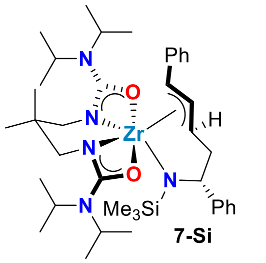

7-Si

Figure S5. HMBC NMR spectrum of 7-Si (C_6D_6, 400 MHz, 298 K).

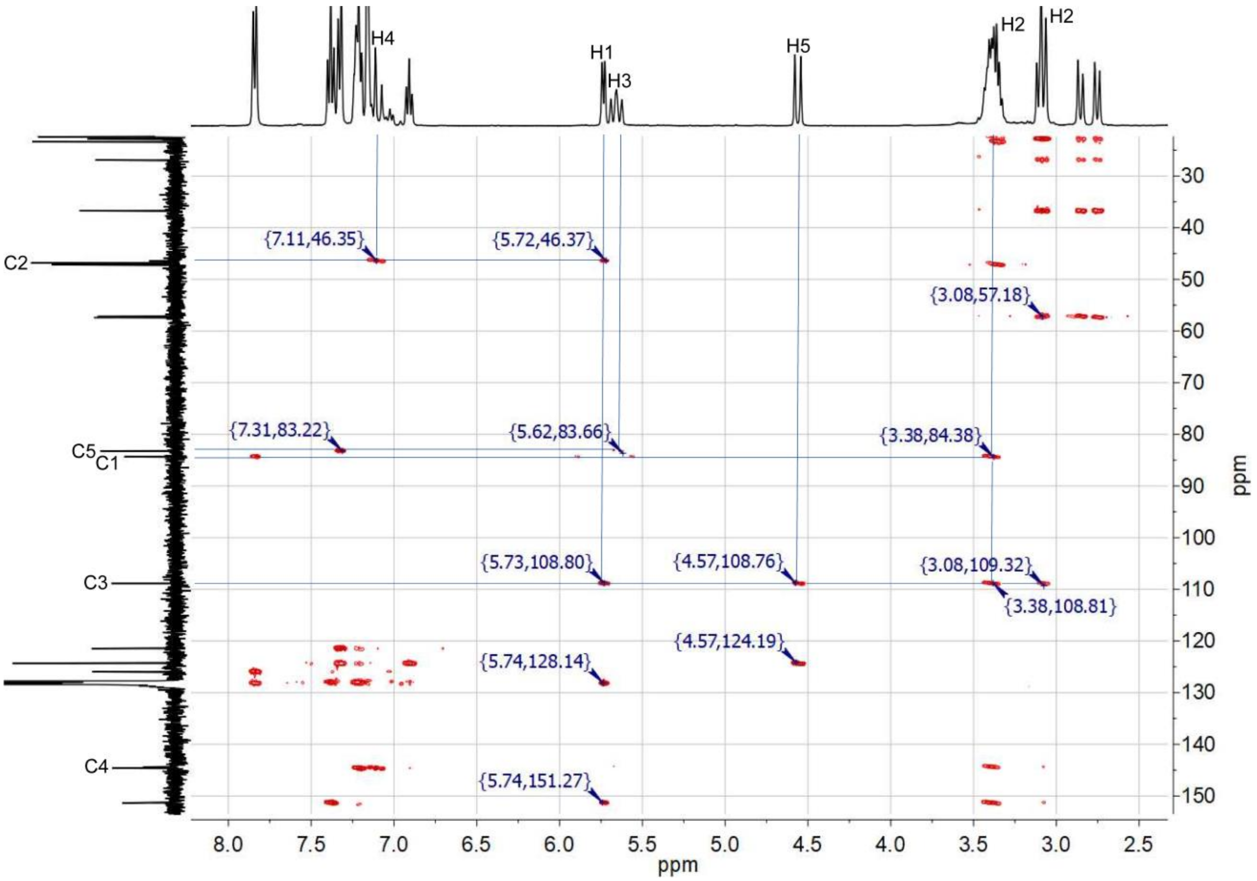

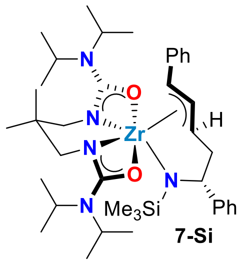

7-Si

Figure S6. ^{1}**H NMR spectrum of 7-Ph (**C_6D_6**, 400 MHz, 298 K).**

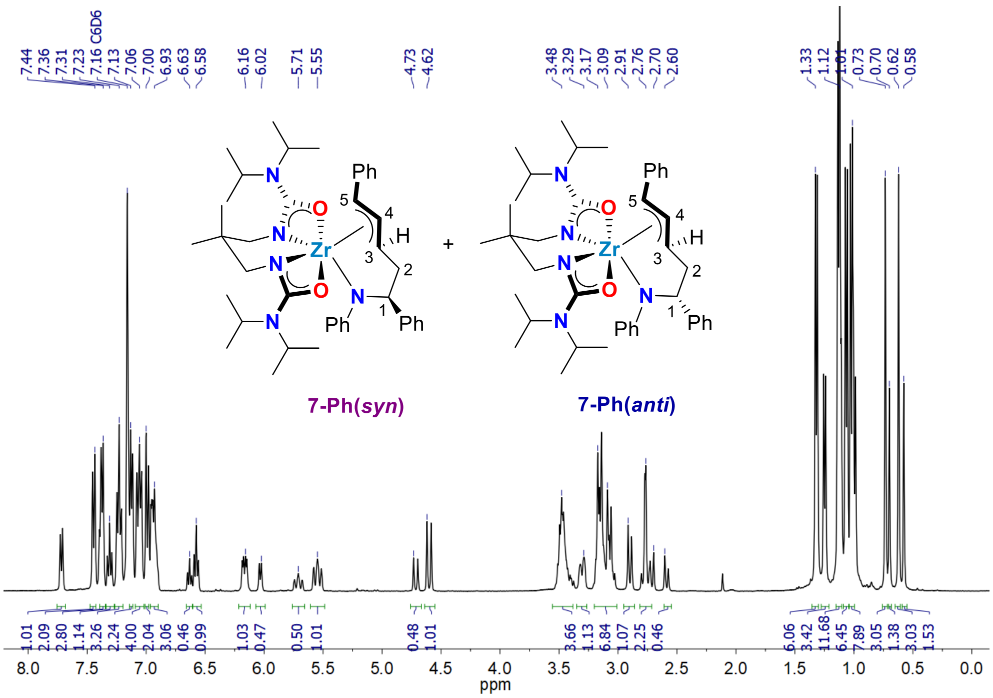

Figure S7. ^{13}**C{**^{1}**H} NMR spectrum of 7-Ph (**C_6D_6**, 100 MHz, 298 K).**

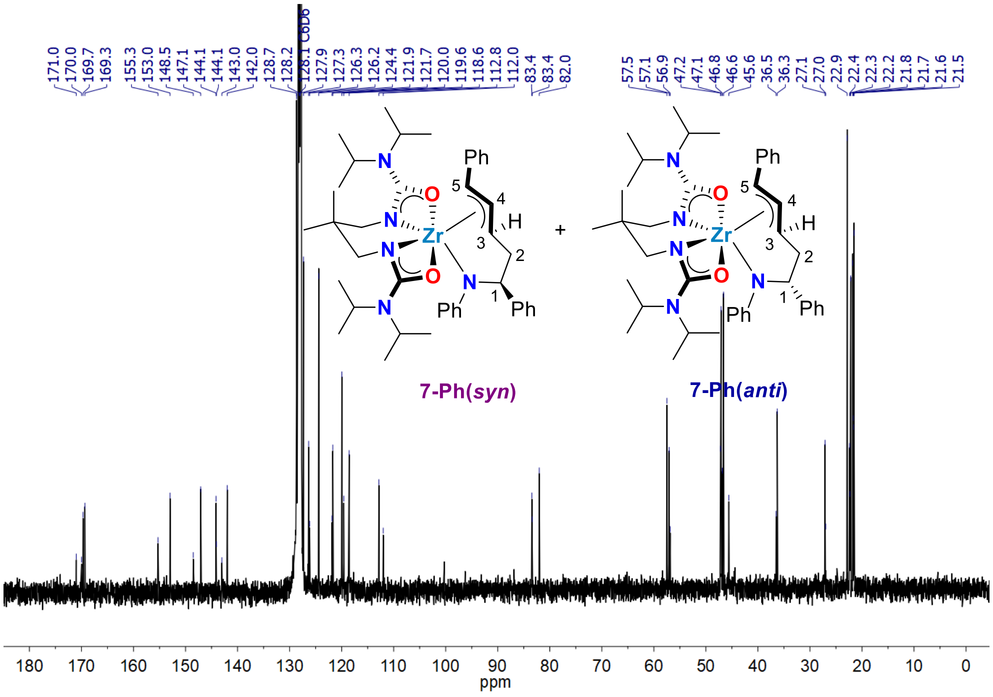

Figure S8. COSY NMR spectrum of 7-Ph (C_6D_6, 400 MHz, 298 K).

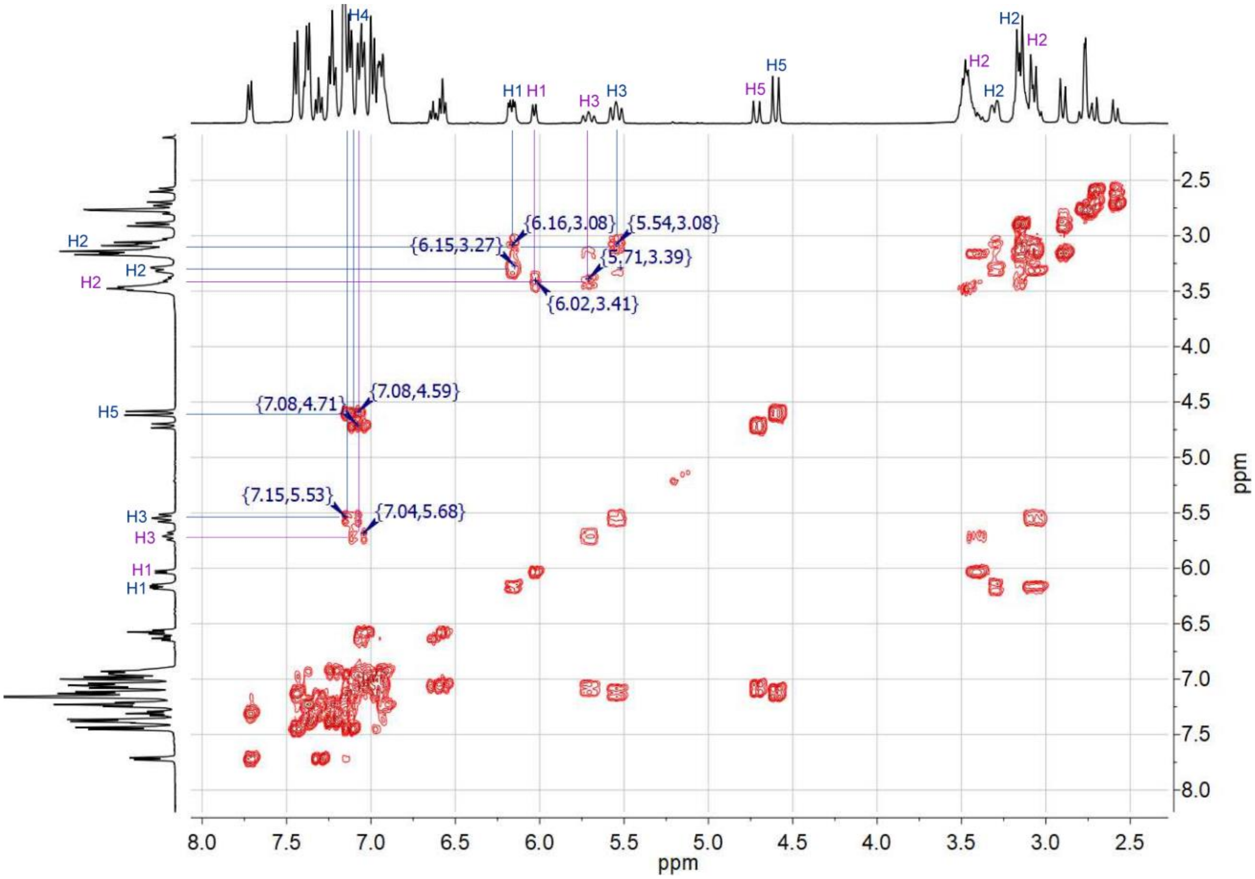

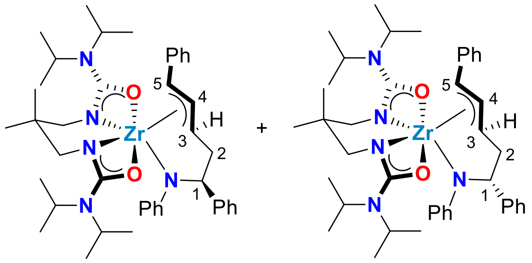

7-Ph(*syn*) **7-Ph(*anti*)**

Figure S9. HSQC NMR spectrum of 7-Ph (C_6D_6, 400 MHz, 298 K).

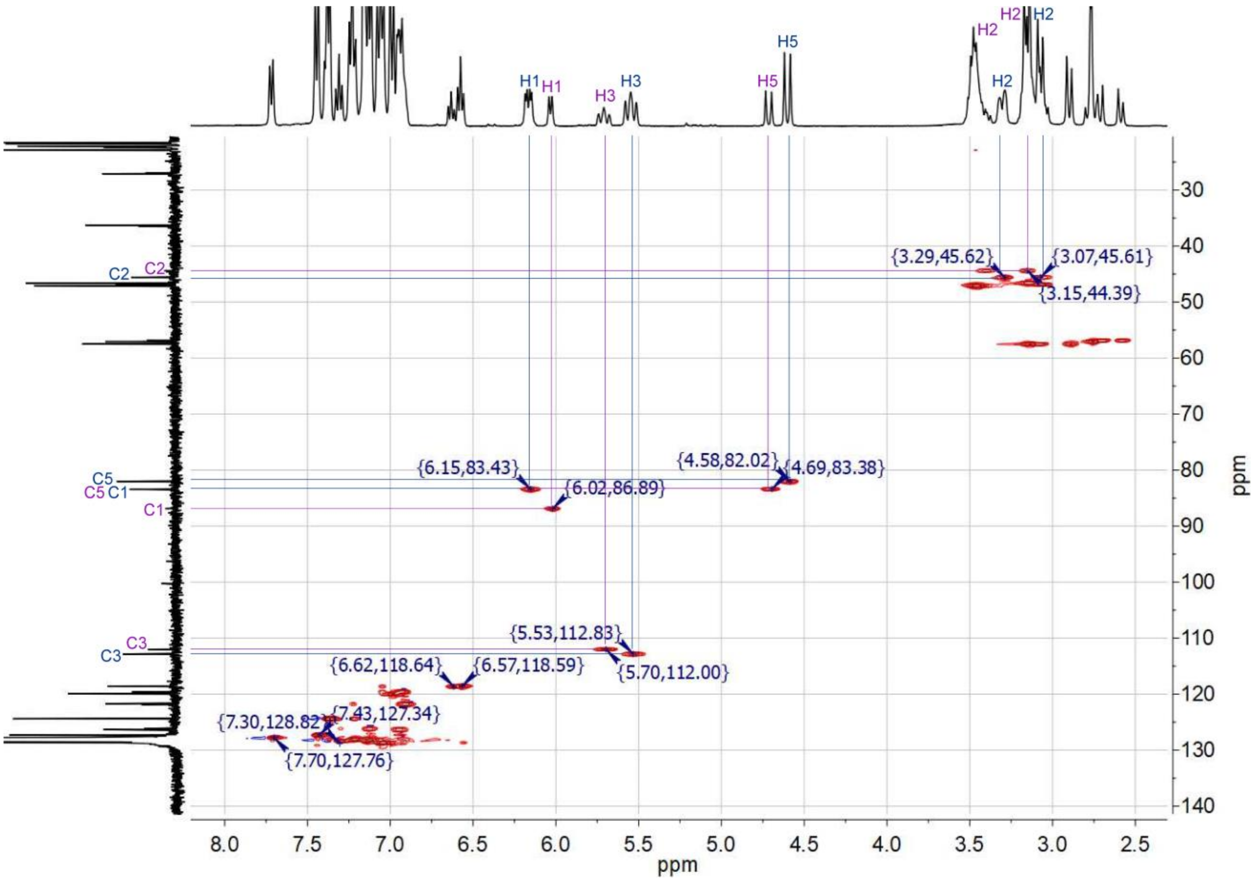

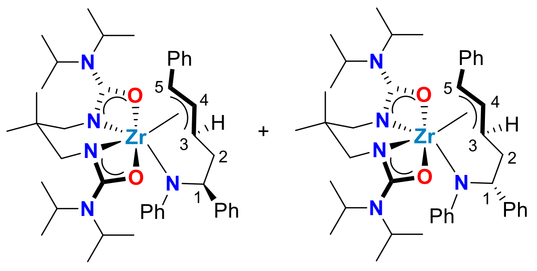

7-Ph(*syn*) **7-Ph(*anti*)**

Figure S10. HMBC NMR spectrum of 7-Ph (C_6D_6, 400 MHz, 298 K).

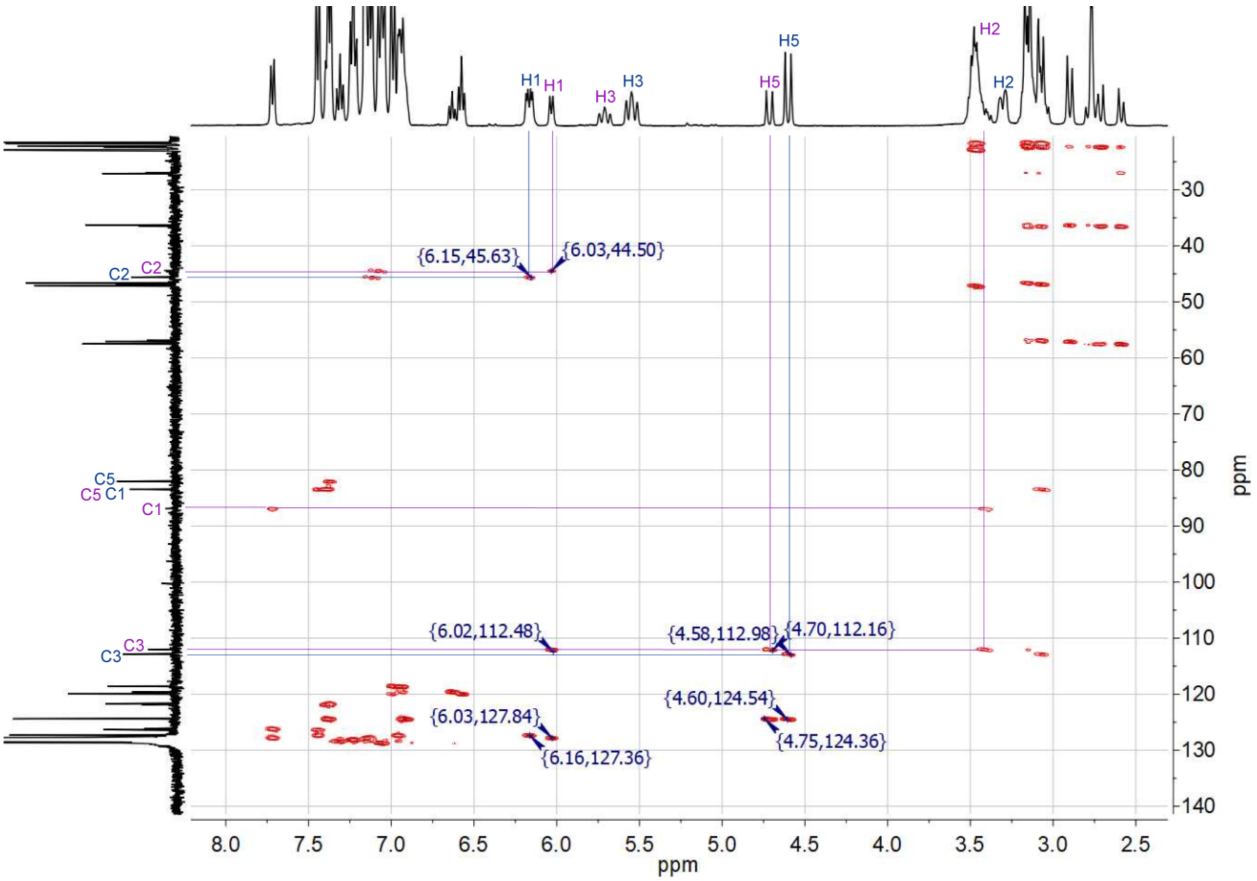

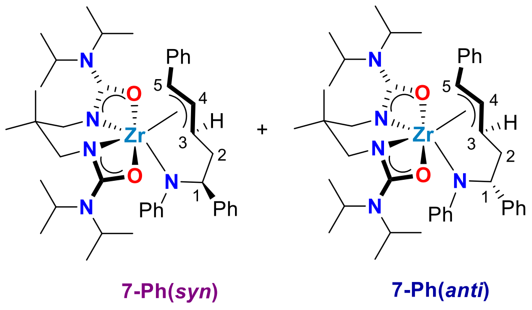

Figure S11. ^{1}**H NMR spectrum of 8-Ph (**C_6D_6**, 400 MHz, 298 K).**

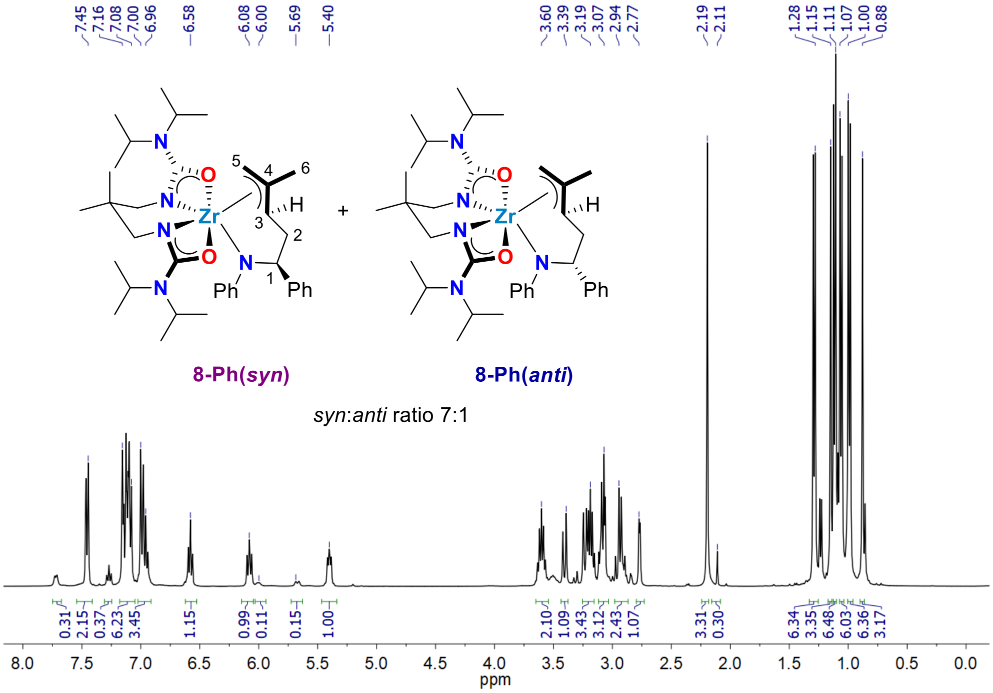

Figure S12. ^{13}**C{**^{1}**H} NMR spectrum of 8-Ph (**C_6D_6**, 100 MHz, 298 K).**

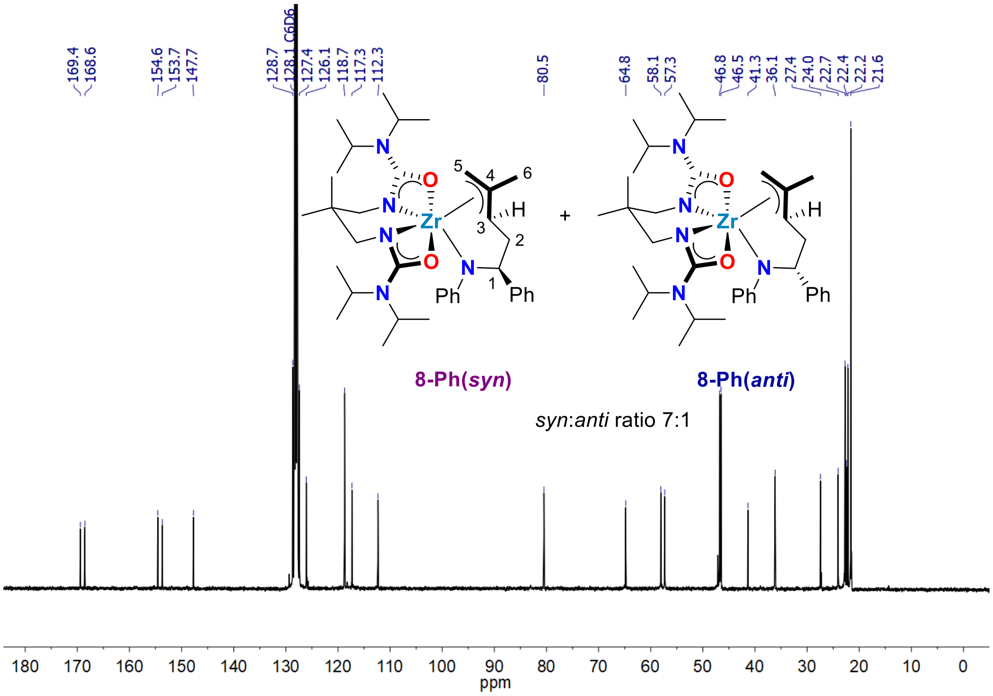

Figure S13. COSY NMR spectrum of 8-Ph (C_6D_6, 400 MHz, 298 K).

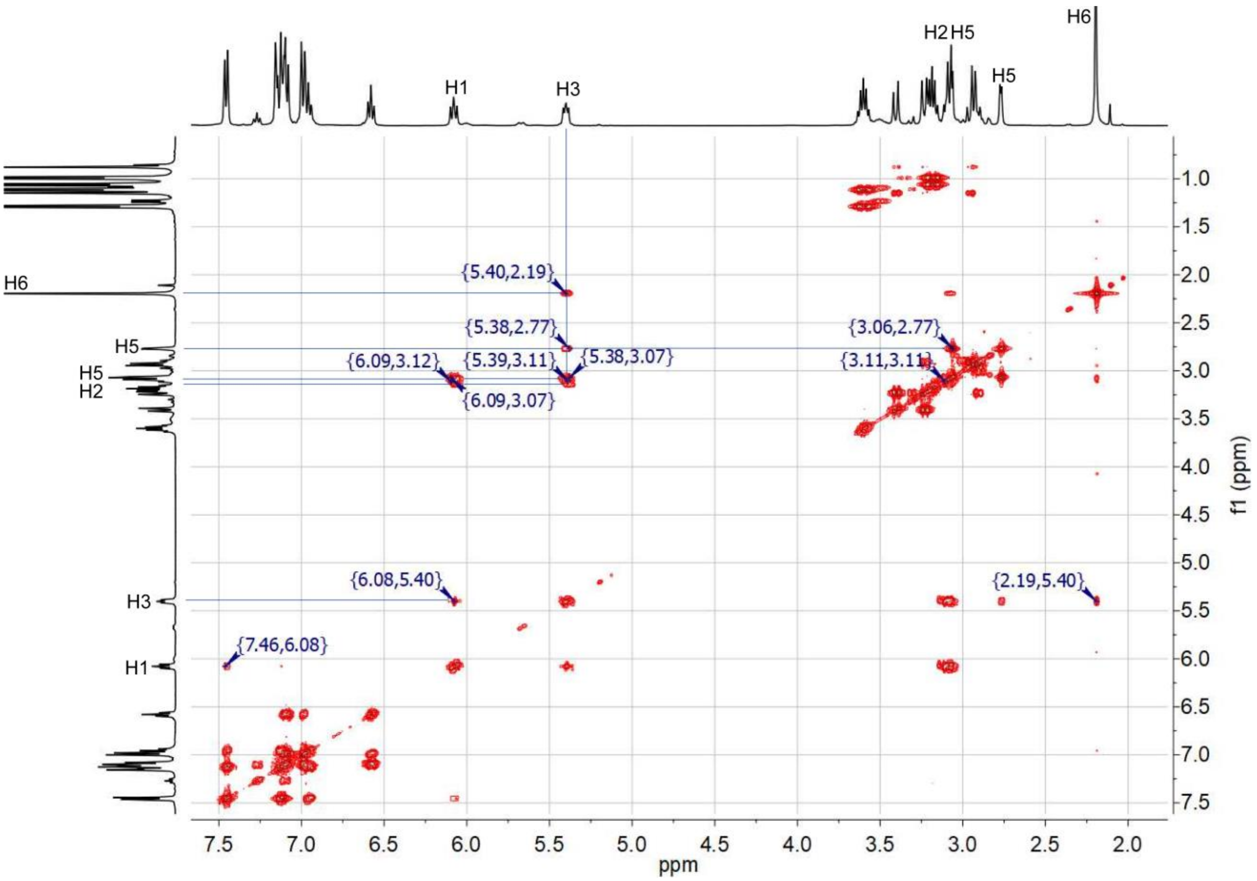

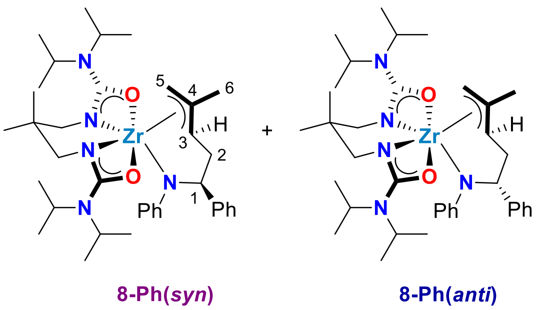

syn:*anti* ratio 7:1

Figure S14. HSQC NMR spectrum of 8-Ph (C_6D_6, 400 MHz, 298 K).

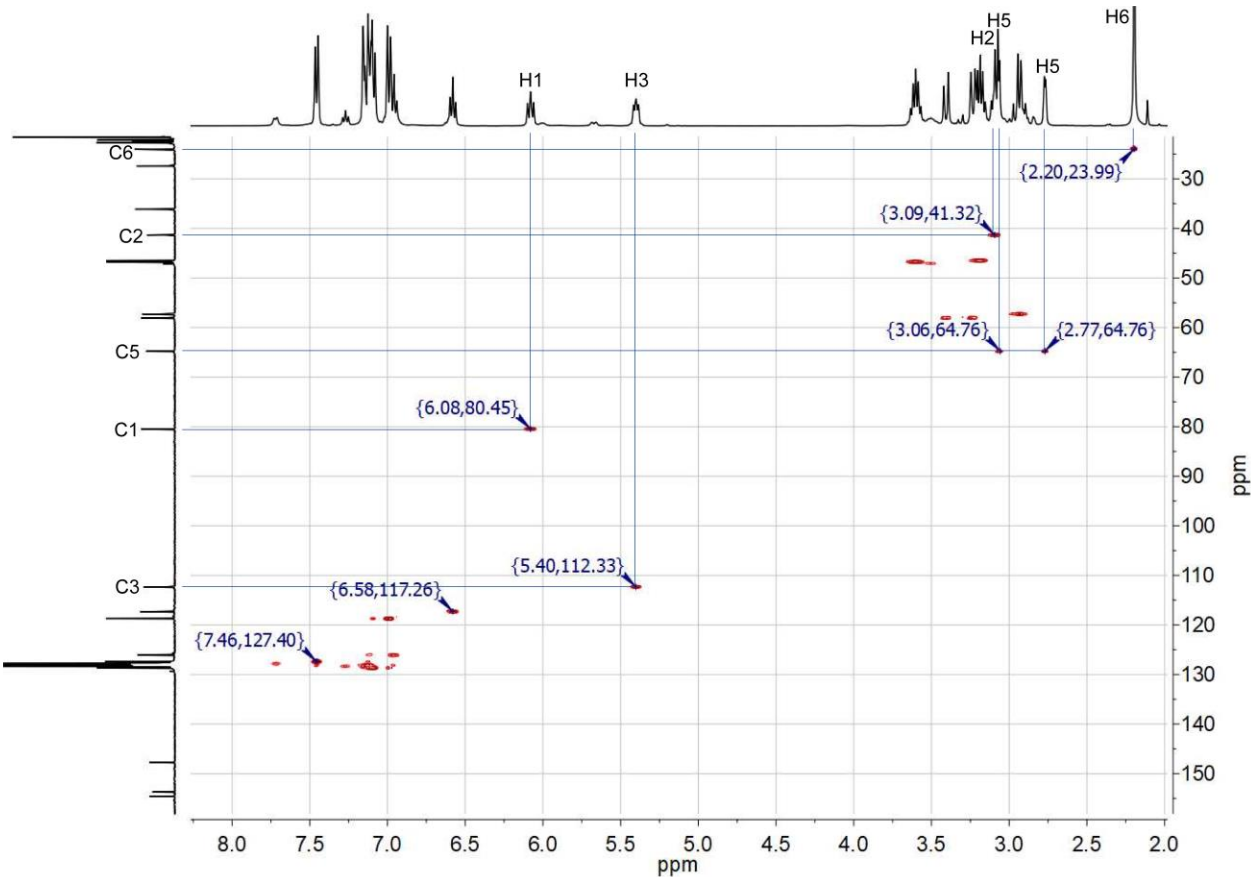

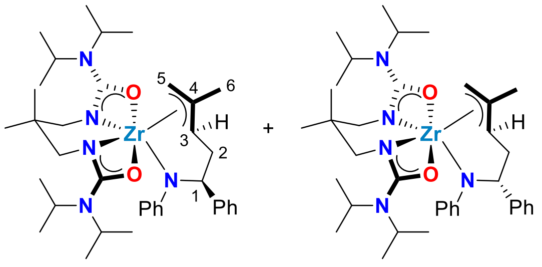

8-Ph(*syn*) **8-Ph(*anti*)**

syn:*anti* ratio 7:1

Figure S15. HMBC NMR spectrum of 8-Ph (C_6D_6, 400 MHz, 298 K).

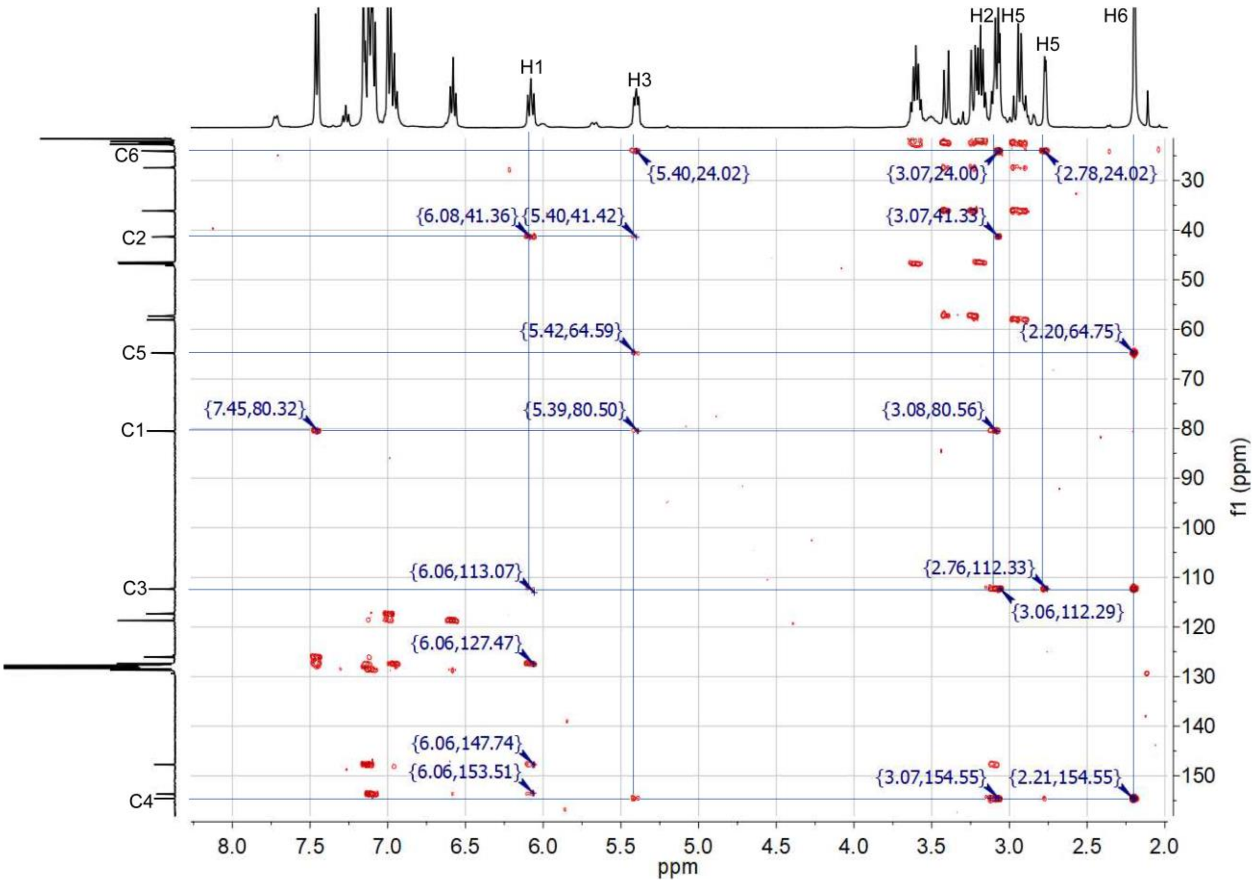

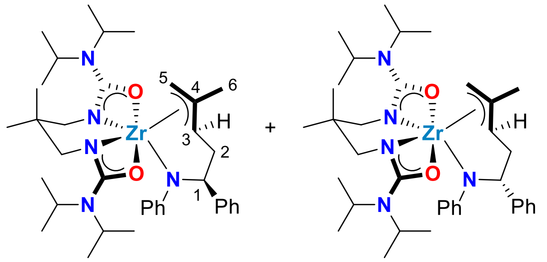

8-Ph(*syn*) **8-Ph(*anti*)**

syn:*anti* ratio 7:1

Figure S16. ^{1}H NMR spectrum of (*E*)-1 ($CDCl_3$, 400 MHz, 298 K).

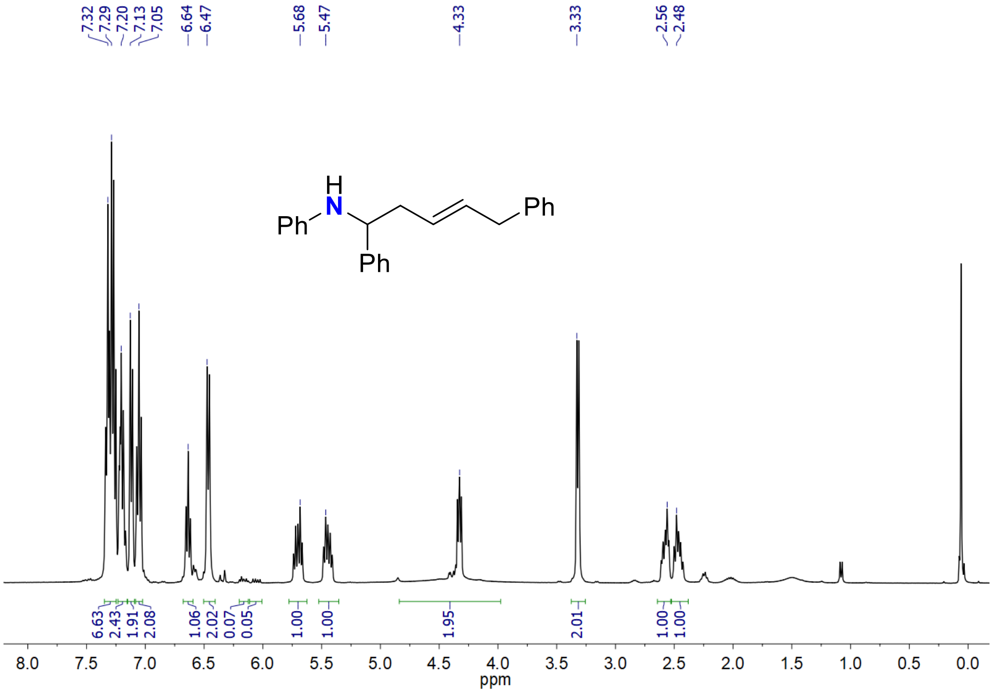

Figure S17. ^{1}H NMR spectrum of (*E*)-2 ($CDCl_3$, 400 MHz, 298 K).

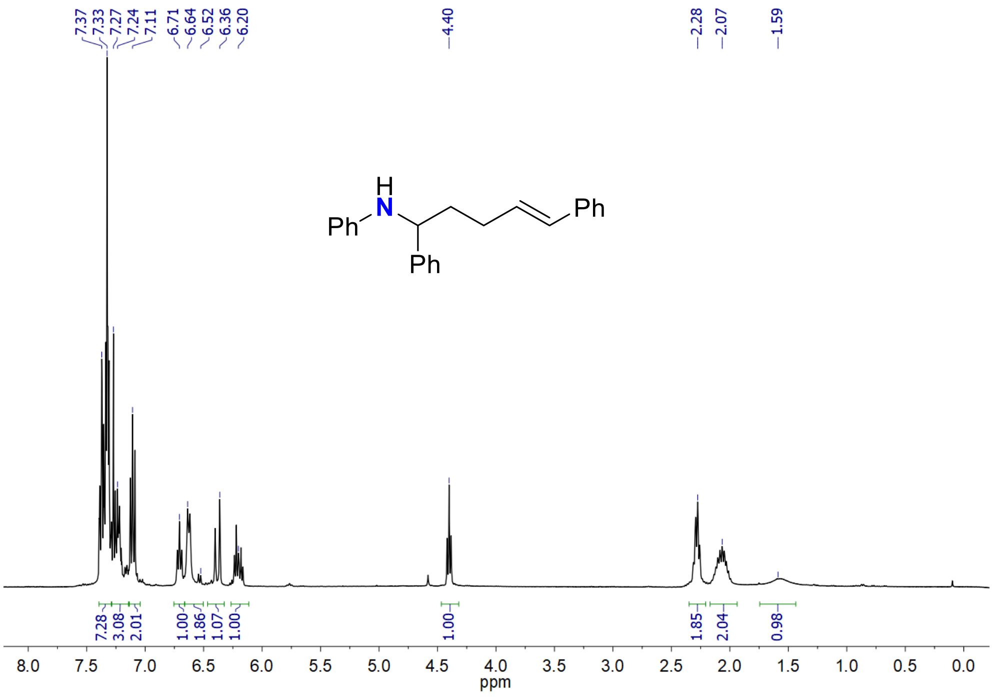

Figure S18. $^{13}C\{^1H\}$ **NMR spectrum of (*E*)-2 ($CDCl_3$, 100 MHz, 298 K).**

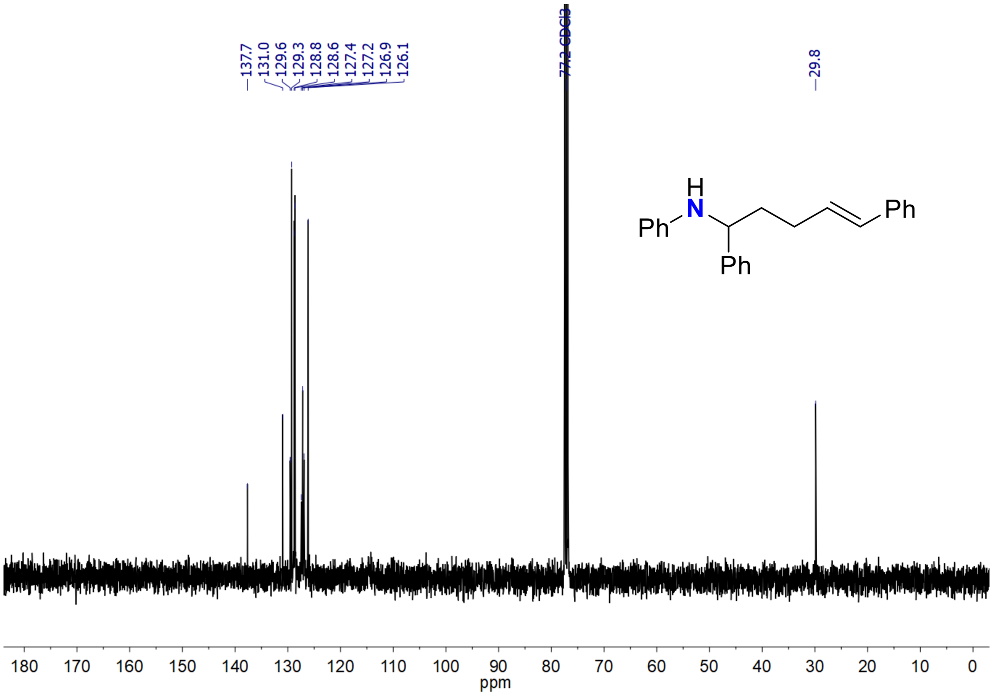

Figure S19. COSY NMR spectrum of (*E*)-2 ($CDCl_3$, 400 MHz, 298 K).

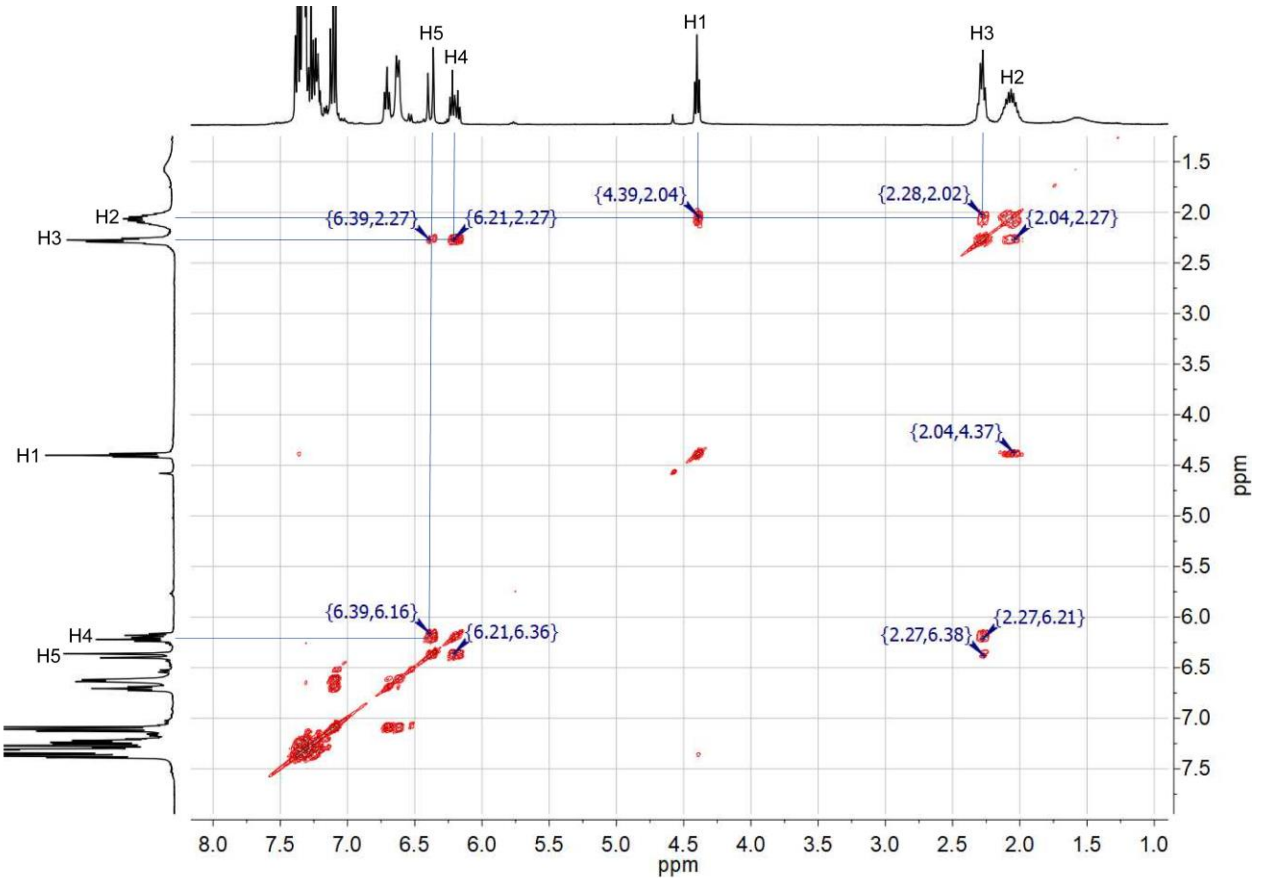

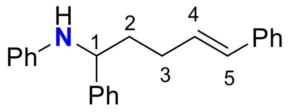

Figure S20. HSQC NMR spectrum of (*E*)-2 ($CDCl_3$, 400 MHz, 298 K).

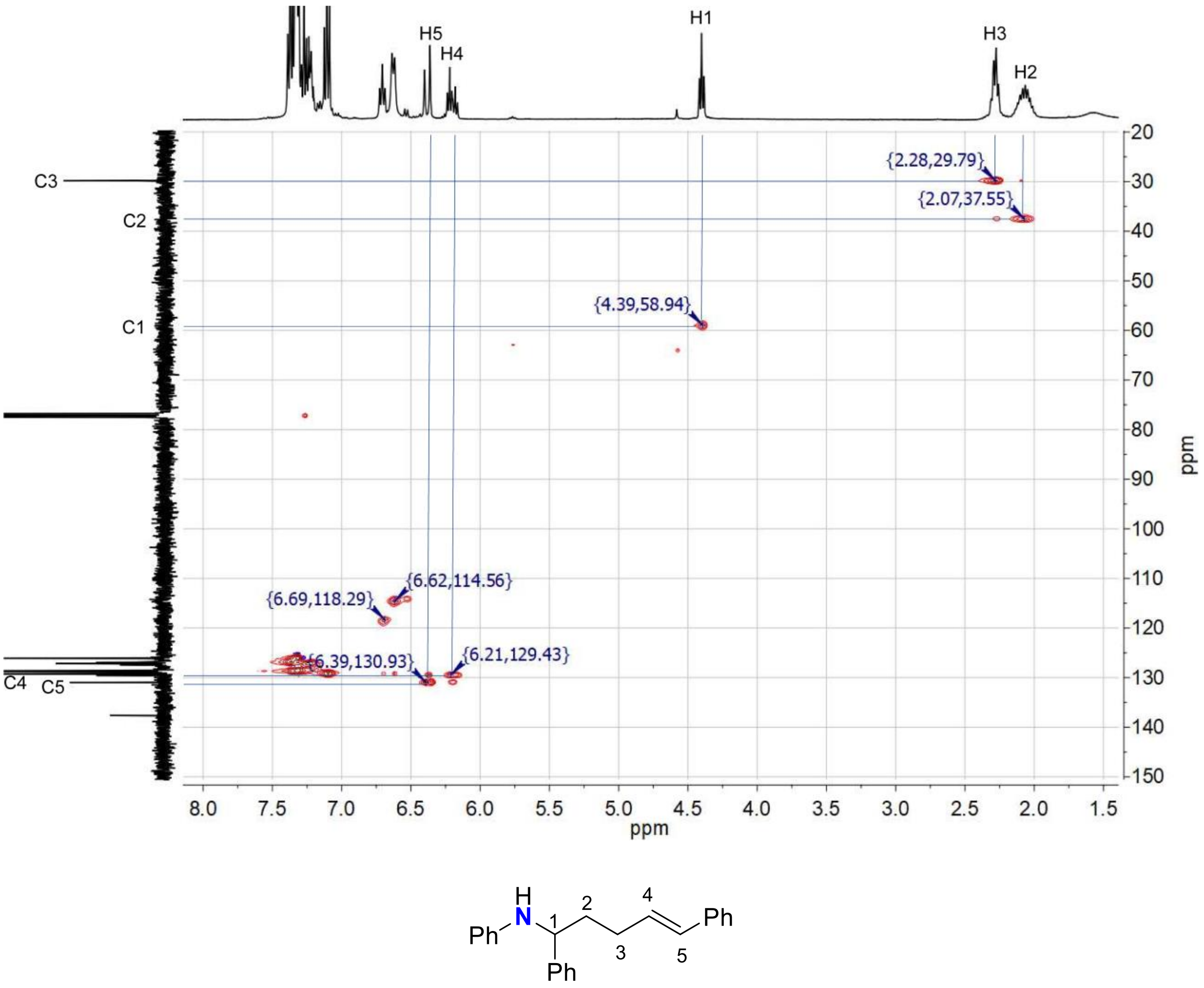

Figure S21. ^{1}H NMR spectrum of (*E*)-4 ($CDCl_3$, 400 MHz, 298 K).

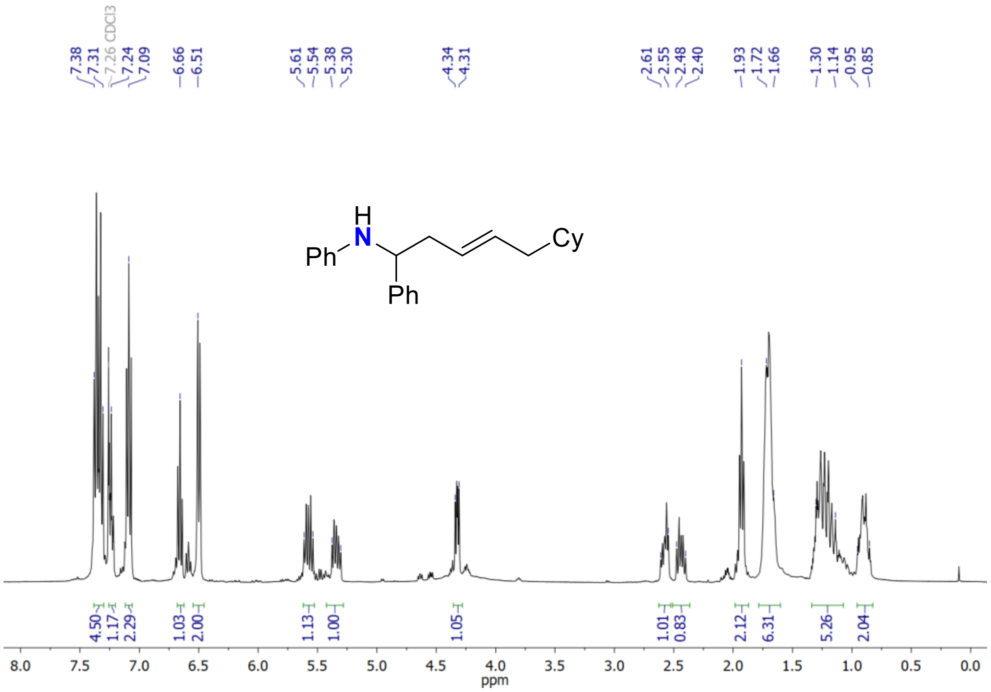

Figure S22. $^{13}C\{^{1}H\}$ NMR spectrum of (*E*)-4 ($CDCl_3$, 100 MHz, 298 K).

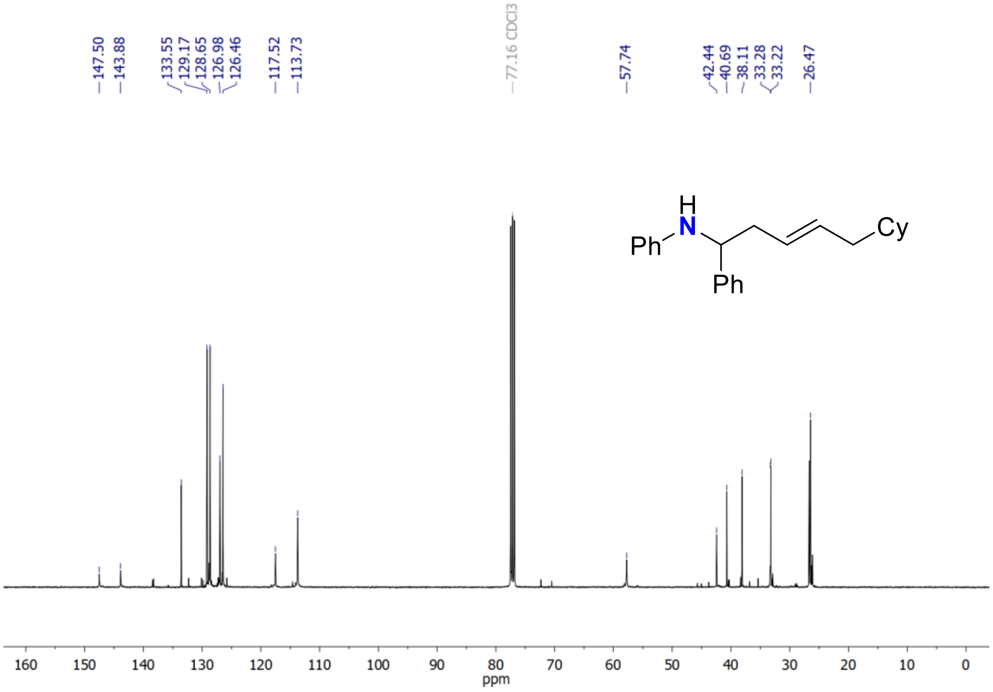

Figure S23. COSY NMR spectrum of (*E*)-4 ($CDCl_3$, 400 MHz, 298 K).

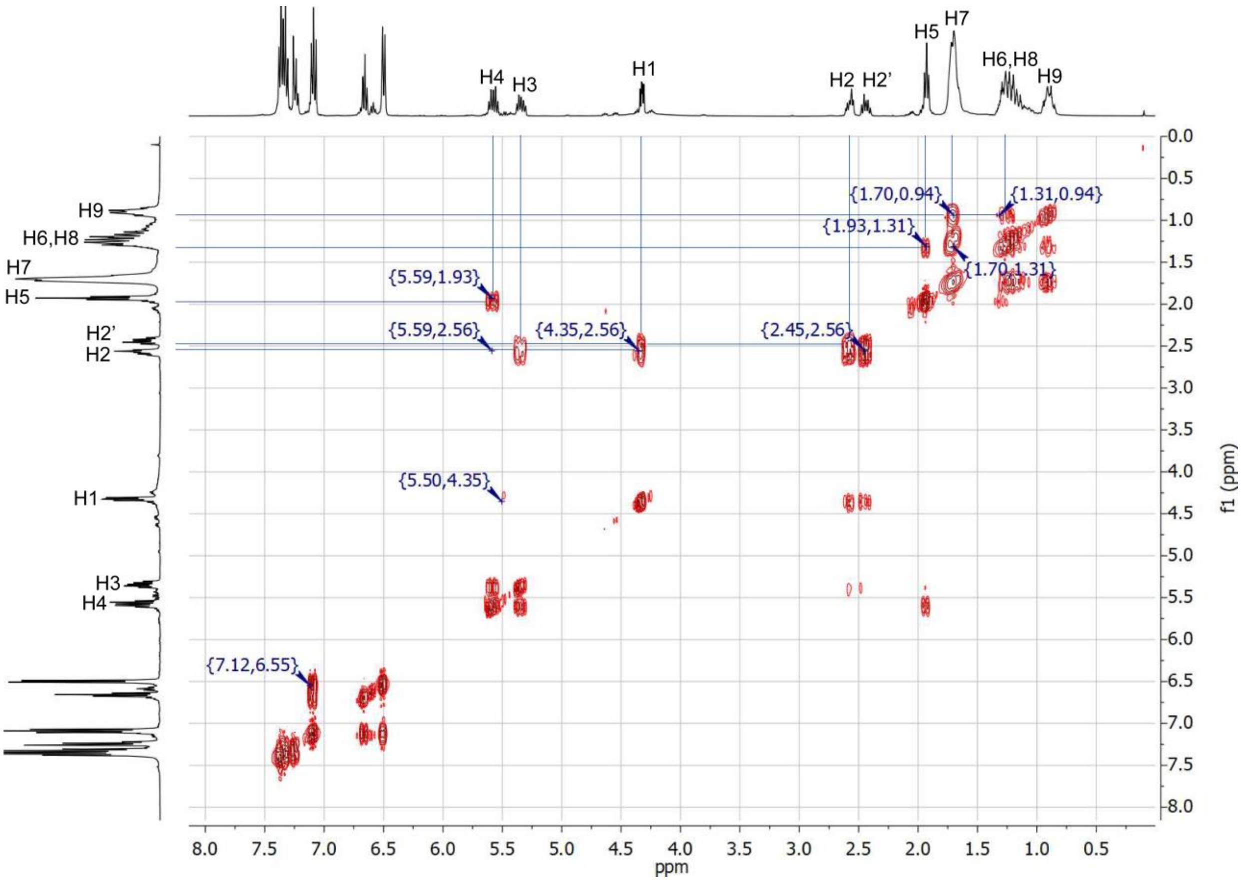

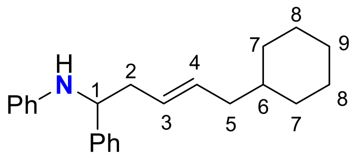

Figure S24. HSQC NMR spectrum of (*E*)-4 ($CDCl_3$, 400 MHz, 298 K).

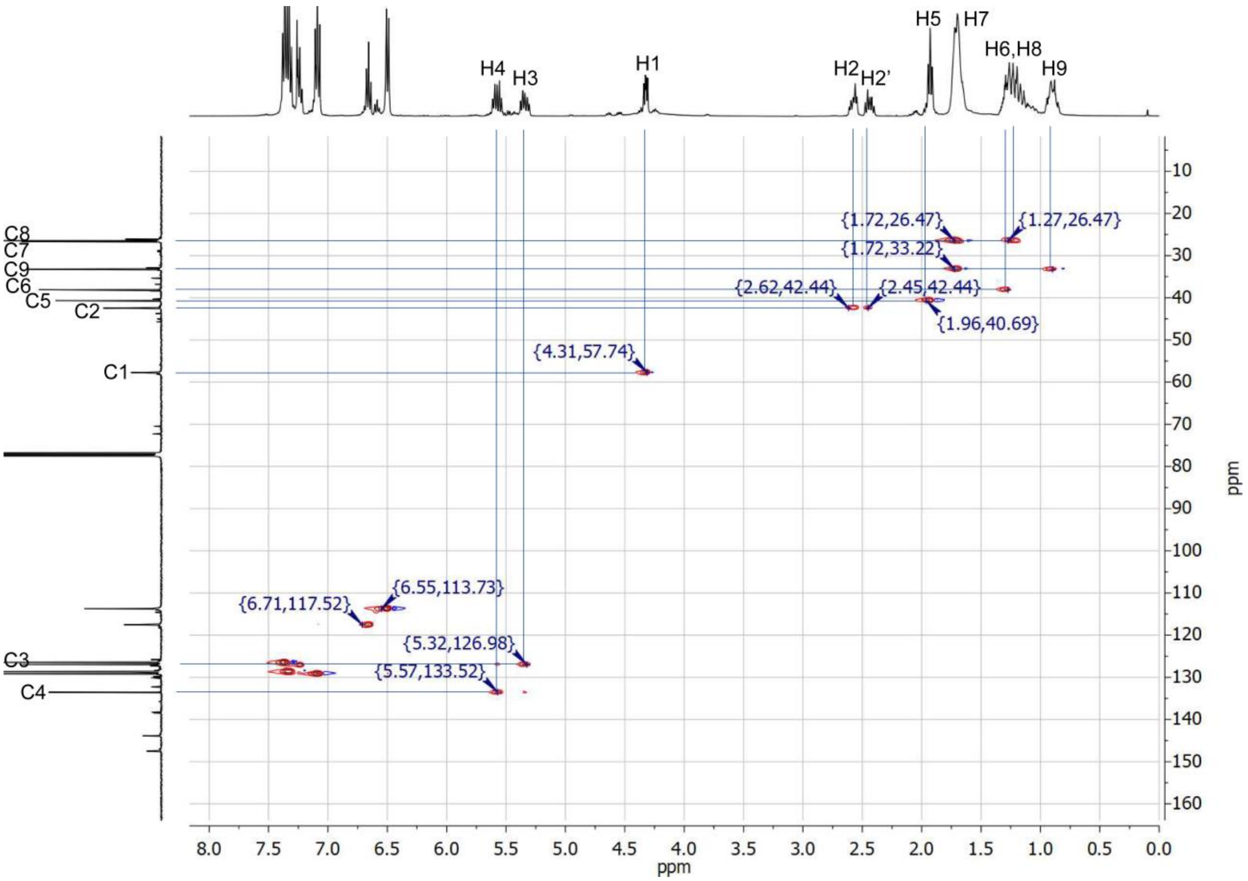

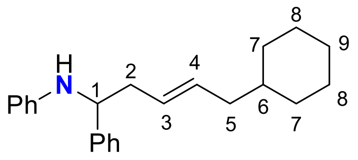

Figure S25. ^{1}H NMR spectrum of 5 ($CDCl_3$, 400 MHz, 298 K).

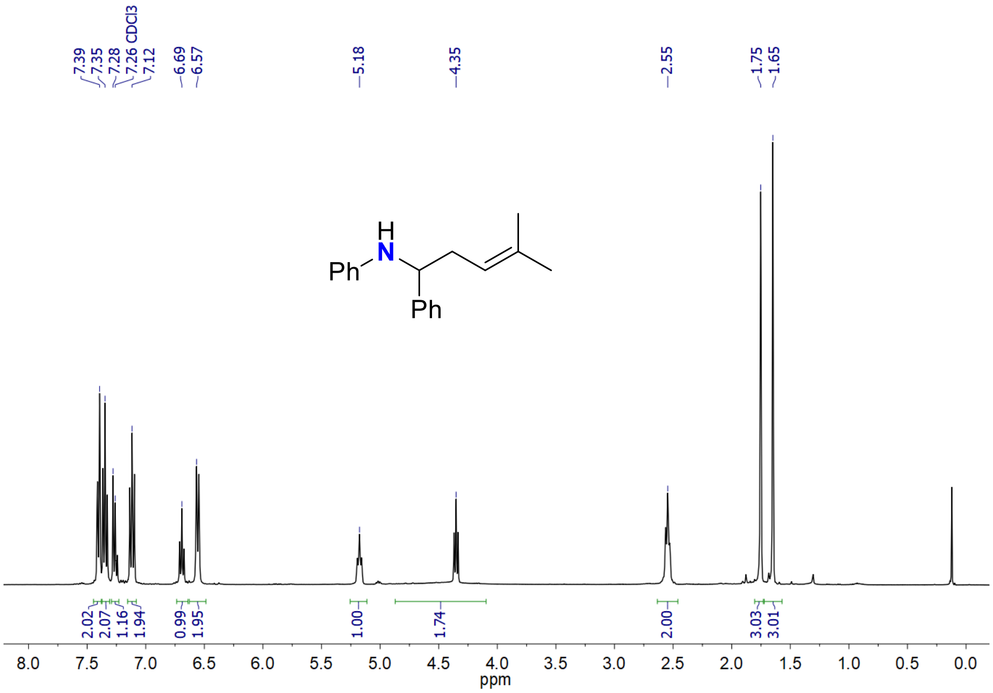

Figure S26. $^{13}C\{^{1}H\}$ NMR spectrum of 5 ($CDCl_3$, 100 MHz, 298 K).

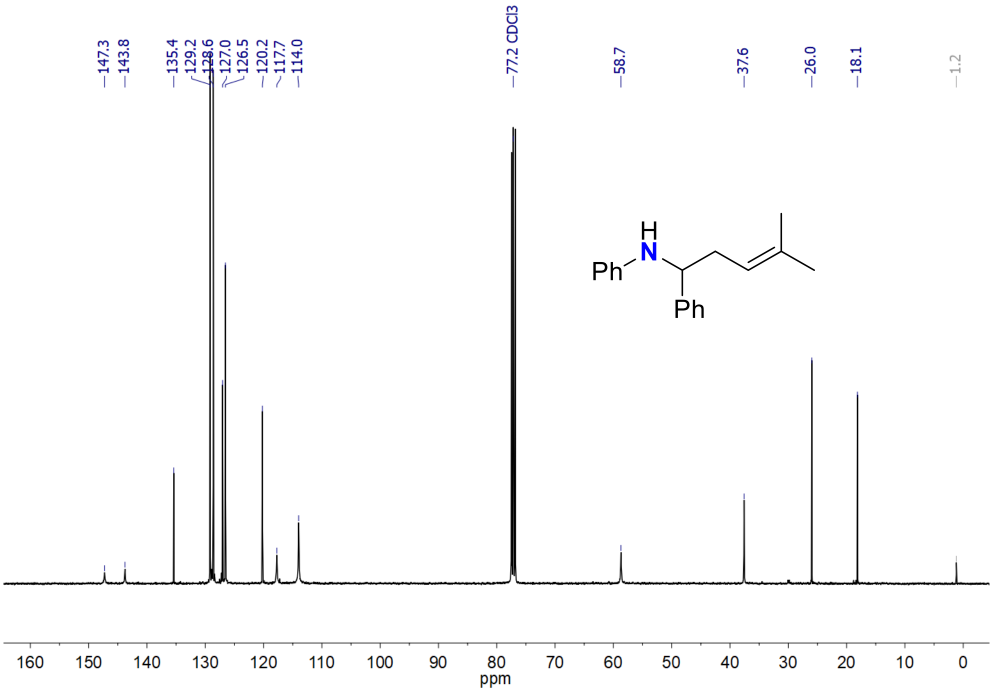

Figure S27. ^{1}H NMR of the catalytic reaction with 1-phenyl-1,3-butadiene catalyzed by *in situ* formed bis(ureate) zirconium catalyst (C_6D_6, 400 MHz, 298 K). Diagnostic resonances: 1,2,3,4-tetramethylbenzene, δ = δ = 6.86 ppm and 2.09 ppm; olefinic CHs δ = 5.43 ppm and 5.28 ppm; benzylic CH of (*E*)-1, δ = 4.21 ppm (Resonance overlapping with benzylic CH of (*E*)-2).

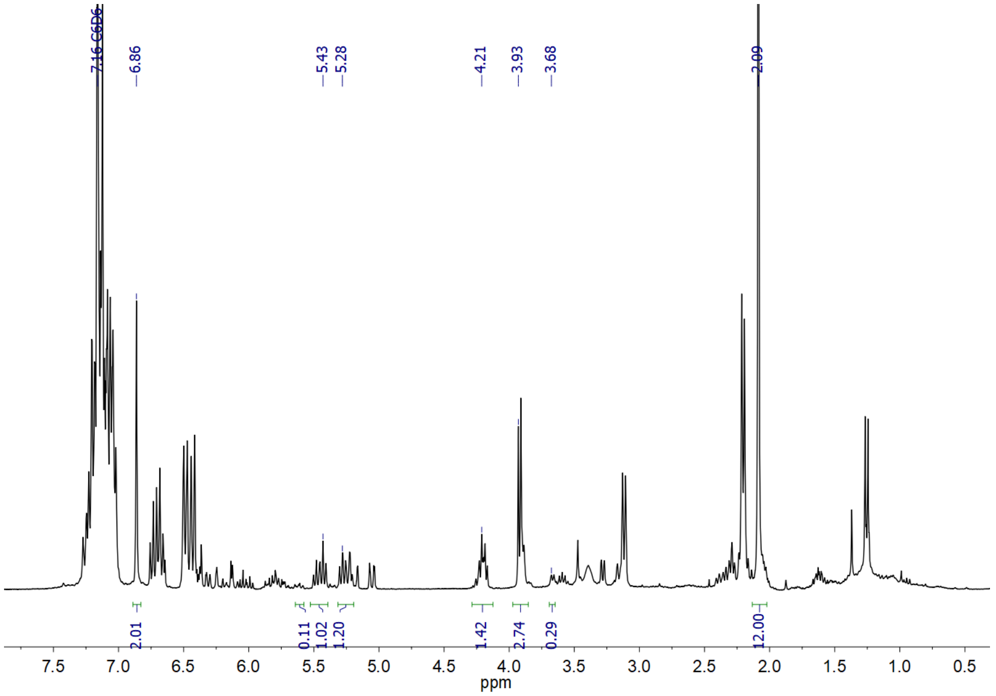

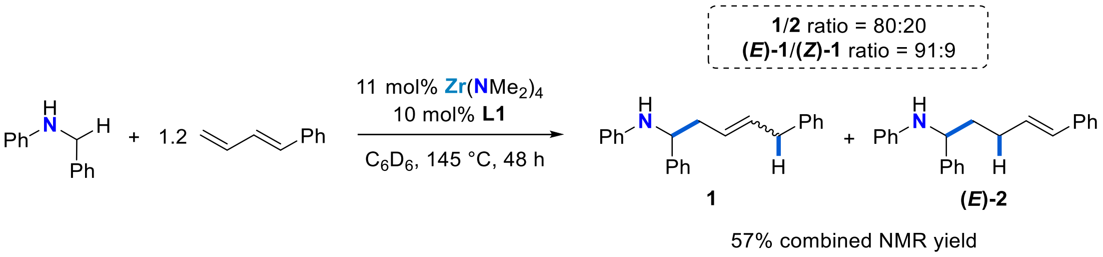

Figure S28. ^{1}H **NMR of the Ti-catalyzed reaction with 1-phenyl-1,3-butadiene (C_6D_6, 400 MHz, 298 K). Diagnostic resonances: 1,3,5-trimethoxybenzene, δ = 6.25 ppm and 3.32 ppm; benzylic CH of (*E*)-2, δ = 4.21 ppm; NH of (*E*)-2, δ = 3.68 ppm; benzylic CH_2 of unreacted *N*-benzylaniline, 3.91 ppm.**

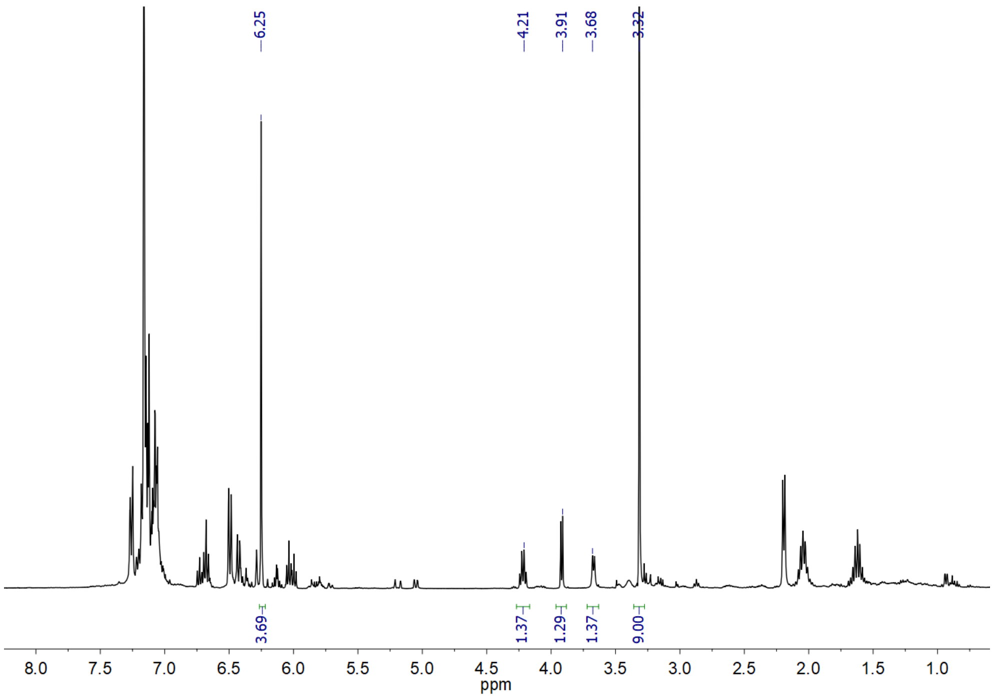

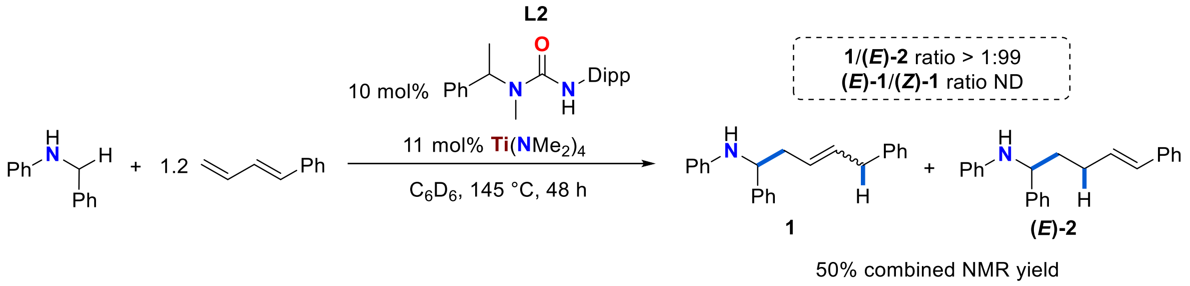

Figure S29. ¹H NMR of the catalytic reaction with N-(trimethylsilyl)benzylamine catalyzed by *in situ* formed bis(ureate) zirconium catalyst (C_6D_6, 400 MHz, 298 K). Diagnostic resonances: 1,2,3,4-tetramethylbenzene, *δ* = *δ* = 6.86 ppm; olefin CH's of 3, *δ* = 5.48 and 5.37 ppm; benzylic CH of 3, *δ* = 3.89 ppm; benzylic CH_2 of unreacted *N*-benzylaniline, *δ* = 3.80 ppm.

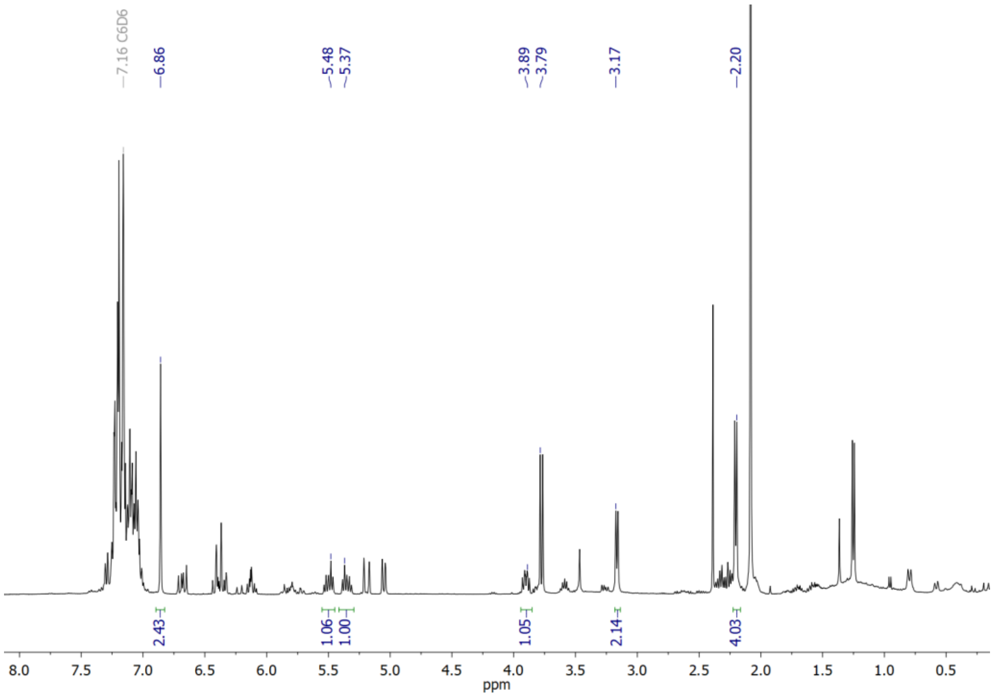

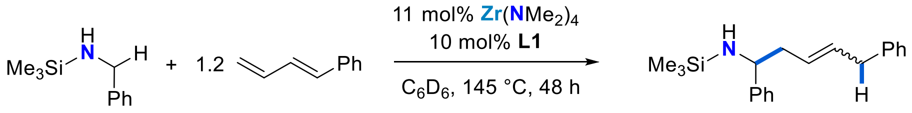

3

Figure S30. ^{1}H NMR of the catalytic reaction with 1-cyclohexyl-1,3-butadiene catalyzed by *in situ* formed bis(ureate) zirconium catalyst (C_6D_6, 400 MHz, 298 K). Diagnostic resonances: 1,3,5-trimethoxybenzene, δ = 6.24 ppm and 3.33 ppm; olefin CH's of (*E*)-4, δ = 5.35 and 5.26 ppm; benzylic CH of (*E*)-4, δ = 4.22 ppm; NH of (*E*)-4, δ = 3.98 ppm; benzylic CH_2 of unreacted *N*-benzylaniline, δ = 3.91 ppm.

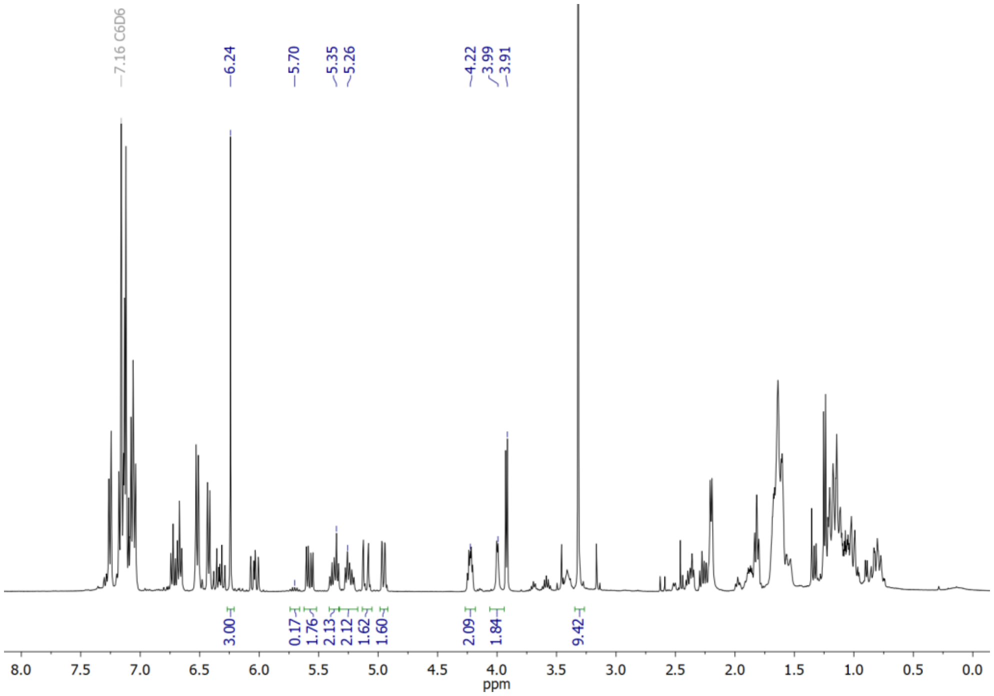

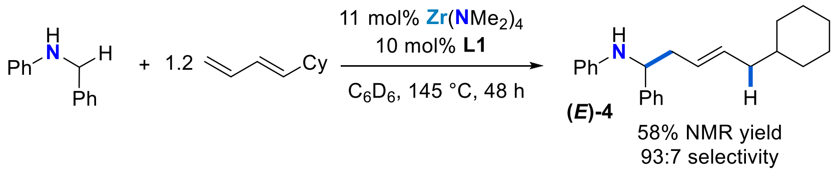

Figure S31. **^{1}H NMR of the catalytic reaction with isoprene catalyzed by *in situ* formed bis(ureate) zirconium catalyst (C_6D_6, 400 MHz, 298 K). Diagnostic resonances: 1,3,5-trimethoxybenzene, δ = 6.25 ppm and 3.33 ppm; benzylic CH of 5, δ = 4.25 ppm; benzylic CH_2 of unreacted *N*-benzylaniline, 3.94 ppm (resonance overlapping with NH of 5).**

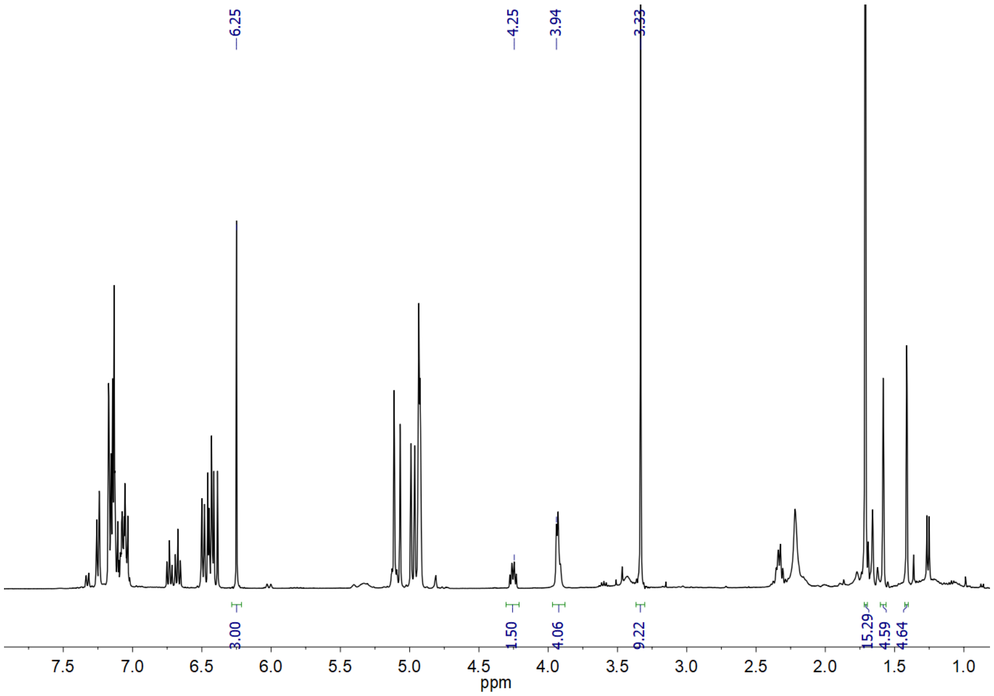

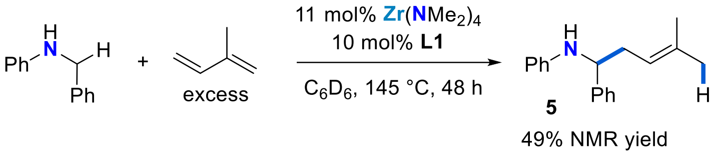

Figure S32. ^{1}**H NMR of the crossover reaction between complex 8-Ph and 5 equivalents of 1-phenyl-1,3-butadiene resulting in the formation of 7-Ph(*syn*) and 7-Ph(*anti*). (C_6D_6, 400 MHz, 298 K). Diagnostic resonances: H5 of 7-Ph(*syn*), *δ* = 4.73; H5 of 7-Ph(*anti*), *δ* = 4.62; H3 of 8-Ph, *δ* = 5.41 ppm; methyl groups of isoprene, *δ* = 1.70 ppm.**

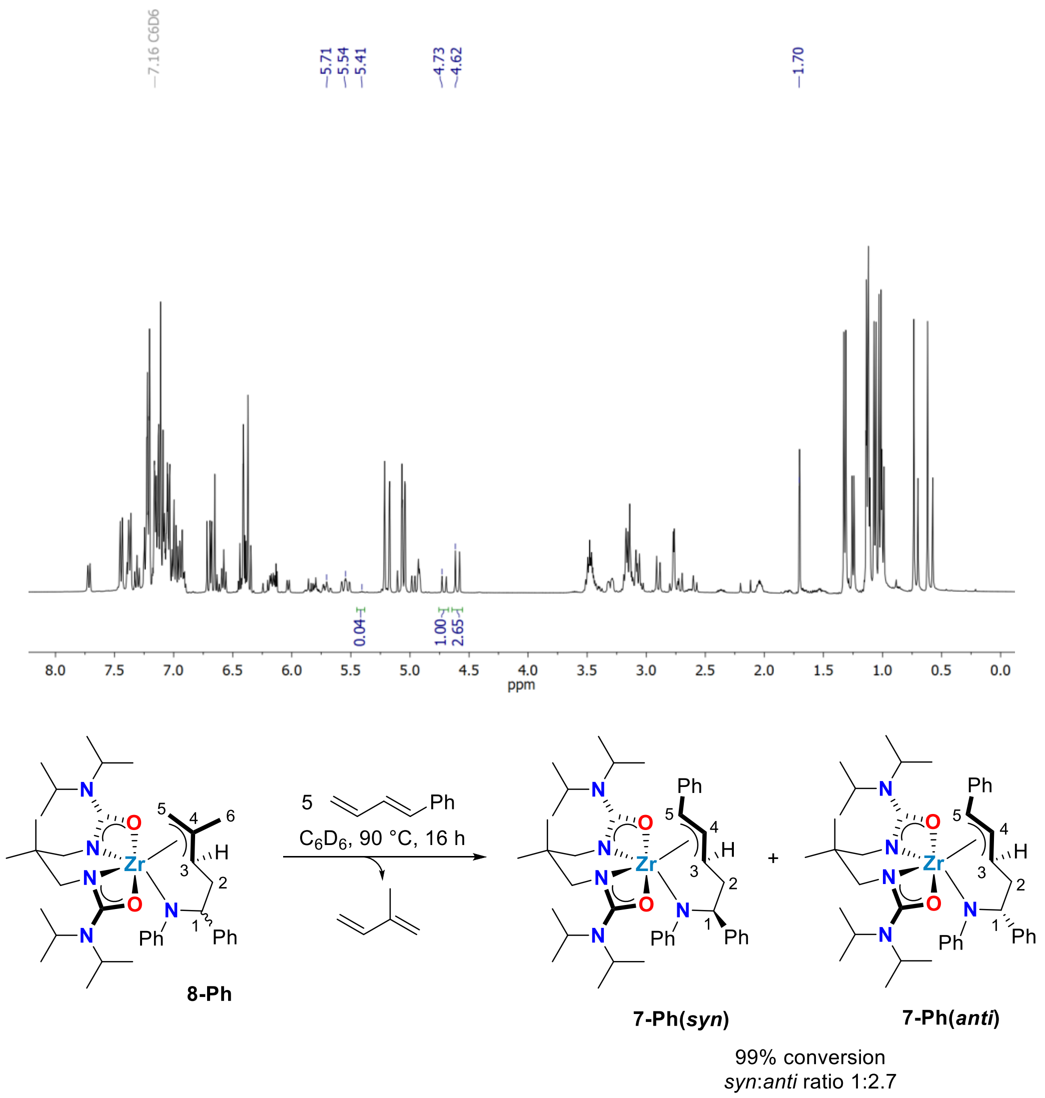

Crystallographic details

A summary of the crystallographic data for compounds **7-Si**, **7-Ph(*syn*)**, and **8-Ph(*syn*)** is shown in Table S1. The automatic data collection strategy, indexing and integration were carried out using the Bruker APEX3 Crystallography Software Suite. Using *Olex2*,[8] the structures were solved with the *ShelXT*[9] structure solution program using Intrinsic Phasing and the structures were refined using the *ShelXL*[10] refinement package using the Least Squares method.

Table S1. List of crystallographic parameters for compounds 7-Si, 7-Ph(*syn*), and 8-Ph(*syn*).

Compound	**7-Si**	**7-Ph(*syn*)**
Empirical formula	$C_{39}H_{63}N_5O_2SiZr$	$C_{42}H_{59}N_5O_2Zr$
Formula weight	753.28	757.16
Temperature/K	100	100
Crystal system	monoclinic	triclinic
Space group	C12/c1	P-1
a/Å	34.527(4)	10.6003(6)
b/Å	10.5749(13)	13.7655(7)
c/Å	27.352(3)	15.6971(8)
α/°	90	79.490(2)
β/°	111.514(4)	72.182(2)
γ/°	90	67.933(2)
Volume/Å^3	9291(2)	2014.97(19)
Z	8	2
ρ_{calc}g/cm^3	1.209	1.248
μ/mm^{-1}	0.303	0.313
F(000)	3616.0	804.0
Crystal size/mm^3	0.24 × 0.22 × 0.21	0.118 × 0.114 × 0.08
Radiation	MoKα (λ = 0.71073)	MoKα (λ = 0.71073)
2Θ range for data collection/°	3.228 to 61.278	3.202 to 61.136
Index ranges	-14 ≤ h ≤ 14, -19 ≤ k ≤ 19, -20 ≤ l ≤ 20	-15 ≤ h ≤ 15, -19 ≤ k ≤ 19, -22 ≤ l ≤ 22
Reflections collected	14182	59696
Independent reflections	14182 [R_{int} = 0.0661, R_{sigma} = 0.0330]	12351 [R_{int} = 0.0484, R_{sigma} = 0.0425]
Data/restraints/parameters	14182/0/510	12351/0/469
Goodness-of-fit on F^2	1.040	1.012
Final R indexes [I>=2σ (I)]	R_1 = 0.0388, wR_2 = 0.0551	R_1 = 0.0324, wR_2 = 0.0449
Final R indexes [all data]	R_1 = 0.0875, wR_2 = 0.0823	R_1 = 0.0691, wR_2 = 0.0736
Largest diff. peak/hole / e Å^{-3}	0.547/-0.390	0.472/-0.355

Compound	**8-Ph(*syn*)**
Empirical formula	$C_{37}H_{57}N_5O_2Zr$
Formula weight	695.09
Temperature/K	100
Crystal system	triclinic
Space group	P-1
a/Å	10.4694(7)
b/Å	13.7275(10)
c/Å	14.5277(11)
α/°	96.630(2)
β/°	103.052(2)
γ/°	109.933(2)
Volume/Å^3	1869.4(2)
Z	2
ρ_{calc}g/cm^3	1.235
μ/mm^{-1}	0.331
F(000)	740.0
Crystal size/mm^3	0.43 × 0.18 × 0.15
Radiation	MoKα (λ = 0.71073)
2Θ range for data collection/°	3.228 to 61.278
Index ranges	-14 ≤ h ≤ 14, -19 ≤ k ≤ 19, -20 ≤ l ≤ 20
Reflections collected	53872
Independent reflections	11493 [R_{int} = 0.0570, R_{sigma} = 0.0530]
Data/restraints/parameters	11493/0/417
Goodness-of-fit on F^2	1.029
Final R indexes [I>=2σ (I)]	R_1 = 0.0371, wR_2 = 0.0520
Final R indexes [all data]	R_1 = 0.0871, wR_2 = 0.0915
Largest diff. peak/hole / e Å^{-3}	0.887/-0.843

Figure S33. ORTEP representation of complex 7-Si with ellipsoids shown at 50% probability and hydrogen atoms omitted for clarity. Selected bond lengths (Å) and angles (°): Zr1–C5, 2.506(2); Zr1–C4, 2.518(2); Zr1–C3, 2.528(2); Zr1–N1, 2.101(2); C5–C4, 1.412(2); C4–C3, 1.390(2); C5–C4–C3, 122.8(2).

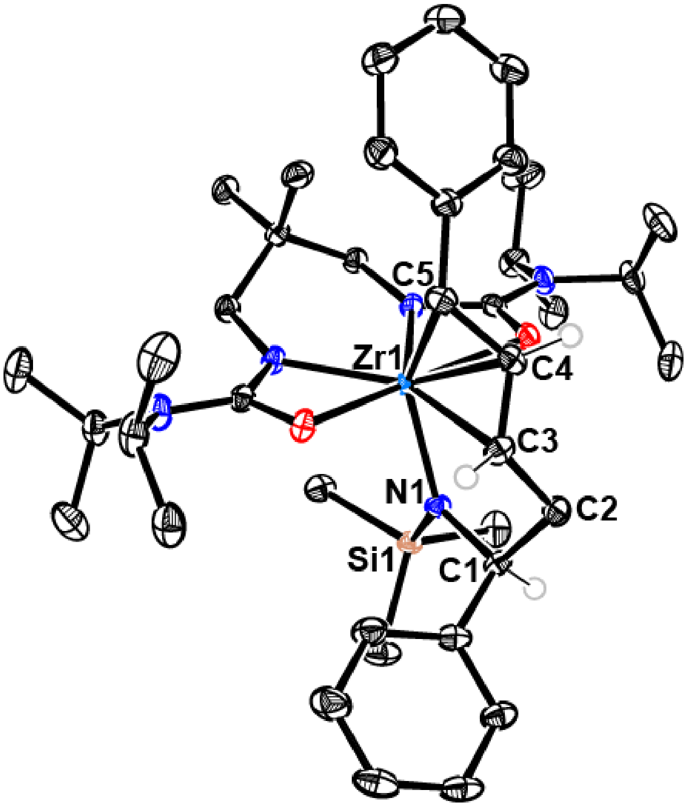

Figure S34. ORTEP representation of complex 7-Ph(*syn*) with ellipsoids shown at 50% probability and hydrogen atoms omitted for clarity. Selected bond lengths (Å) and angles (°): Zr1–C5, 2.493(2); Zr1–C4, 2.514(2); Zr1–C3, 2.519(1); Zr1–N1, 2.126(1); C5–C4, 1.421(2); C4–C3, 1.373(2); C5–C4–C3, 124.5(1).

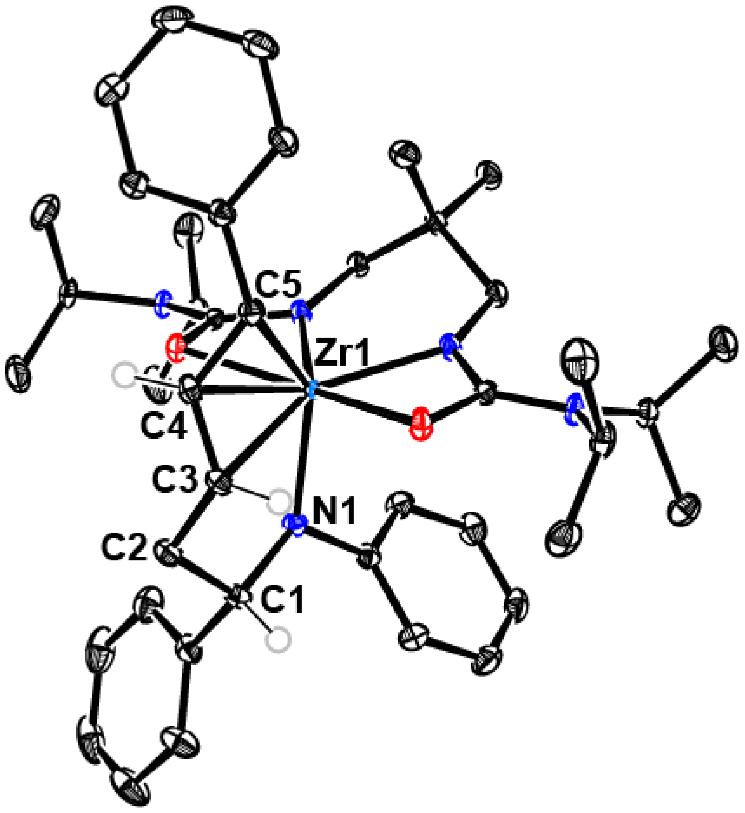

Figure S35. ORTEP representation of allylic complex 8-Ph(*syn*) with ellipsoids shown at 50% probability and hydrogen atoms omitted for clarity. Selected bond lengths (Å) and angles (°): Zr1–C5, 2.4066(18); Zr1–C4, 2.5650(16); Zr1–C3, 2.5222(16); Zr1–N1, 2.1353(15); C5–C4, 1.435(3); C4–C3, 1.376(3); C3–C2, 1.502(3); C2–C1, 1.544(3); C5–C4–C3, 119.1(2).

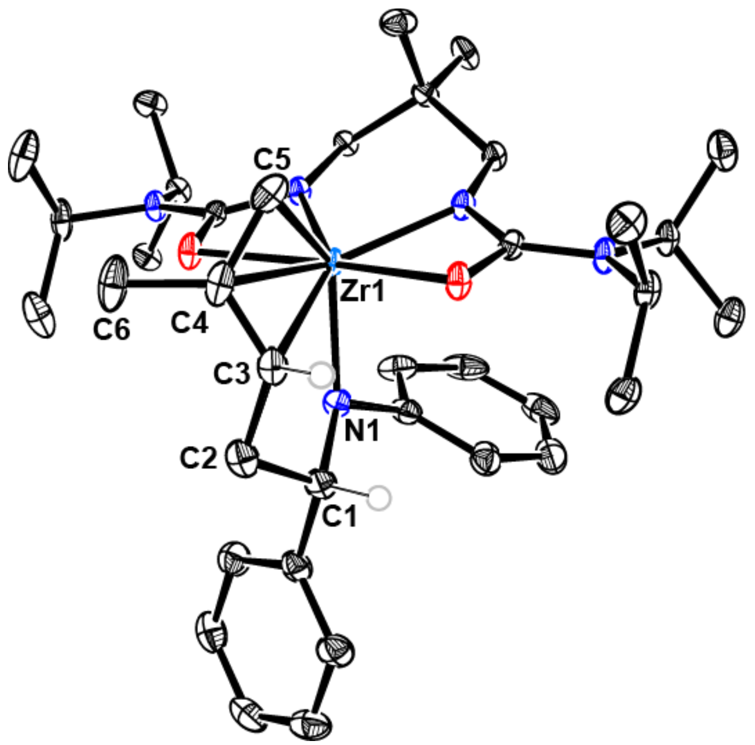

References

1) Leitch, D. C.; Beard, J. D.; Thomson, R. K.; Wright, V. A.; Patrick, B. O.; Schafer, L. L. *Eur. J. Inorg. Chem*. 2009, 2691–2701.

2) Nuñez Bahena, E.; Griffin, S. E.; Schafer, L. L. *J. Am. Chem. Soc.* 2020, **142**, 20566−20571

3) Nuñez Bahena, E.; Patrick, B. O.; Schafer, L. L. *Organometallics.* 2022, **41**, 907−913.

4) DiPucchio, R. C.; Rosca, S. C.; Athavan, G.; Schafer, L. L. *ChemCatChem.* 2019, **11**, 3871–3876.

5) DiPucchio, R. C.; Roşca, S.-C.; Schafer, L. L. *Angew. Chem., Int. Ed.* 2018, **57**, 3469–3472.

6) Moorhouse, S.; Wilkinson, G. *J. Chem. Soc. Dalton Trans.* 1974, **20**, 2187–2190.

7) Y.-Q. Li and S.-L. Shi, *Organometallics*. 2021, **40**, 2345–2353.

8) Dolomanov, O. V.; Bourhis, L. J.; Gildea, R. J.; Howard, J. A. K.; Puschmann, H. *J. Appl. Cryst.* 2009, **42**, 339.

9) Sheldrick, G. M. *Acta Cryst. Sect. A* 2015, **71**, 3.

10) Sheldrick, G. M. *Acta Cryst. Sect. C* 2015, **71**, 3.
